# A bacterial expression cloning screen reveals single-stranded DNA-binding proteins as potent desicco-protectants

**DOI:** 10.1016/j.celrep.2024.114956

**Published:** 2024-11-11

**Authors:** Jonathan D. Hibshman, Courtney M. Clark-Hachtel, Kerry S. Bloom, Bob Goldstein

**Affiliations:** 1 Biology Department, University of North Carolina at Chapel Hill, Chapel Hill, NC 27599, USA; 2 Lineberger Comprehensive Cancer Center, University of North Carolina at Chapel Hill, Chapel Hill, NC 27599, USA; 3 Present address: Department of Biological Sciences, Southern Methodist University, Dallas, TX 75205, USA; 4 Lead contact

## Abstract

Desiccation kills most cells. Some proteins have been identified to help certain cells survive desiccation, but many protein protectants are likely to be unknown. Moreover, the mechanisms ensuring protection of key cellular components are incompletely understood. We devised an expression-cloning approach to discover further protectants. We expressed cDNA libraries from two species of tardigrades in *E. coli*, and we subjected the bacteria to desiccation to select for survivors. Sequencing the populations of surviving bacteria revealed enrichment of mitochondrial single-stranded DNA-binding proteins (mtSSBs) from both tardigrade species. Expression of mtSSBs in bacteria improved desiccation survival as strongly as the best tardigrade protectants known to date. We found that DNA-binding activity of mtSSBs was necessary and sufficient to improve the desiccation tolerance of bacteria. Although tardigrade mtSSBs were among the strongest protectants we found, single-stranded DNA binding proteins in general offered some protection. These results identify single-stranded DNA-binding proteins as potent desicco-protectants.

## In brief

Desiccation is a harsh stress that kills most cells and organisms. Hibshman et al. used an expression-cloning approach to screen for tardigrade proteins that could improve bacterial desiccation survival. They found that mitochondrial single-stranded DNA-binding proteins can significantly improve desiccation survival—an effect that relies on their ability to bind DNA.

## Graphical abstract

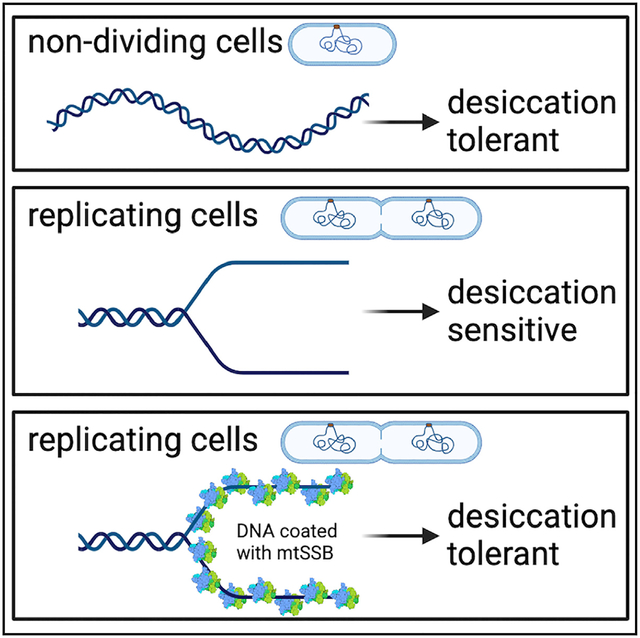

## INTRODUCTION

In 1702, Antonie van Leeuwenhoek described reviving dried “animalcules” from desiccated sediment.^1^ Researchers have since been fascinated with this phenomenon of anhydrobiosis—life without water. Desiccation causes extensive damage to cells, yet some organisms, like those initially observed by van Leeuwenhoek, are able to survive extreme desiccation.^2–5^ Survival in the near-complete absence of cellular water has been reported in some multicellular animals, such as rotifers (as observed by van Leeuwenhoek), nematodes, certain arthropods such as chironomid midges and brine shrimp, and tardigrades.^2^ Desiccation tolerance is also prevalent among plant seeds and, in some instances, like the resurrection plant, also in vegetative tissue.^6^ In addition to multicellular animals and plants, some single-celled organisms can survive desiccation, offering simplified models for understanding the cellular basis of desiccation survival. Desiccation may be a selective pressure that can drive adaptations to other extremes, like the tremendous radiation tolerance of the bacterium *Deinococcus radiodurans*.^7^ Desiccation tolerance has also been linked to pathogenicity of clinical isolates of bacteria.^8^ The identification of effective protectants may contribute to understanding the basic biology of anhydrobiosis and also prove useful for stabilizing biological materials, like medicines, cells, or tissues.^9–11^

Tardigrades, also known as water bears, are recognized for their ability to survive extreme conditions, including desiccation, high levels of radiation, temperature stress, and the vacuum of space.^2,12–20^ The capacity of tardigrades to survive what most animals cannot suggests that they may harbor powerful protectants.^21^ There is great interest in discovering such molecules. Some approaches have effectively identified protectants from tardigrades by isolating heat-soluble proteins that are particularly stable,^22^ identifying chromatin-associated proteins that can protect against DNA damage,^23^ and using the transcriptional response of tardigrades to prioritize candidates.^24^ To be useful for applications in biomedicine, a protectant must have potency alone in diverse, non-native contexts. We were encouraged by the examples of tardigrade cytosolic abundant heat soluble (CAHS) proteins that were sufficient to improve desiccation survival in bacteria and yeast and the damage suppressor (Dsup) protein, which could improve radiation tolerance of human cells.^23,24^ We sought an unbiased screening method to search for protectants that would not rely on limiting criteria like transcriptional regulation during desiccation and that would prioritize candidates with potentially broad applicability beyond tardigrades.

Here, we report the development and implementation of a bacterial expression-cloning approach to screen for proteins from tardigrades that can serve as desicco-protectants in *E. coli*. Imposing selection for desiccation survival on a population of bacteria revealed that mitochondrial single-stranded DNA-binding proteins (mtSSBs) from two species of tardigrades are potent desicco-protectants in replicating cells. The protective ability of mtSSBs in bacteria relies on their binding to DNA, and mtSSBs from diverse organisms offer some degree of protection. We propose a model in which coating a cell’s DNA with protein may limit DNA damage and promote desiccation tolerance.

## RESULTS

### Developing bacterial expression cloning as a functional screening approach to identify desicco-protectants

**Figure 1.**
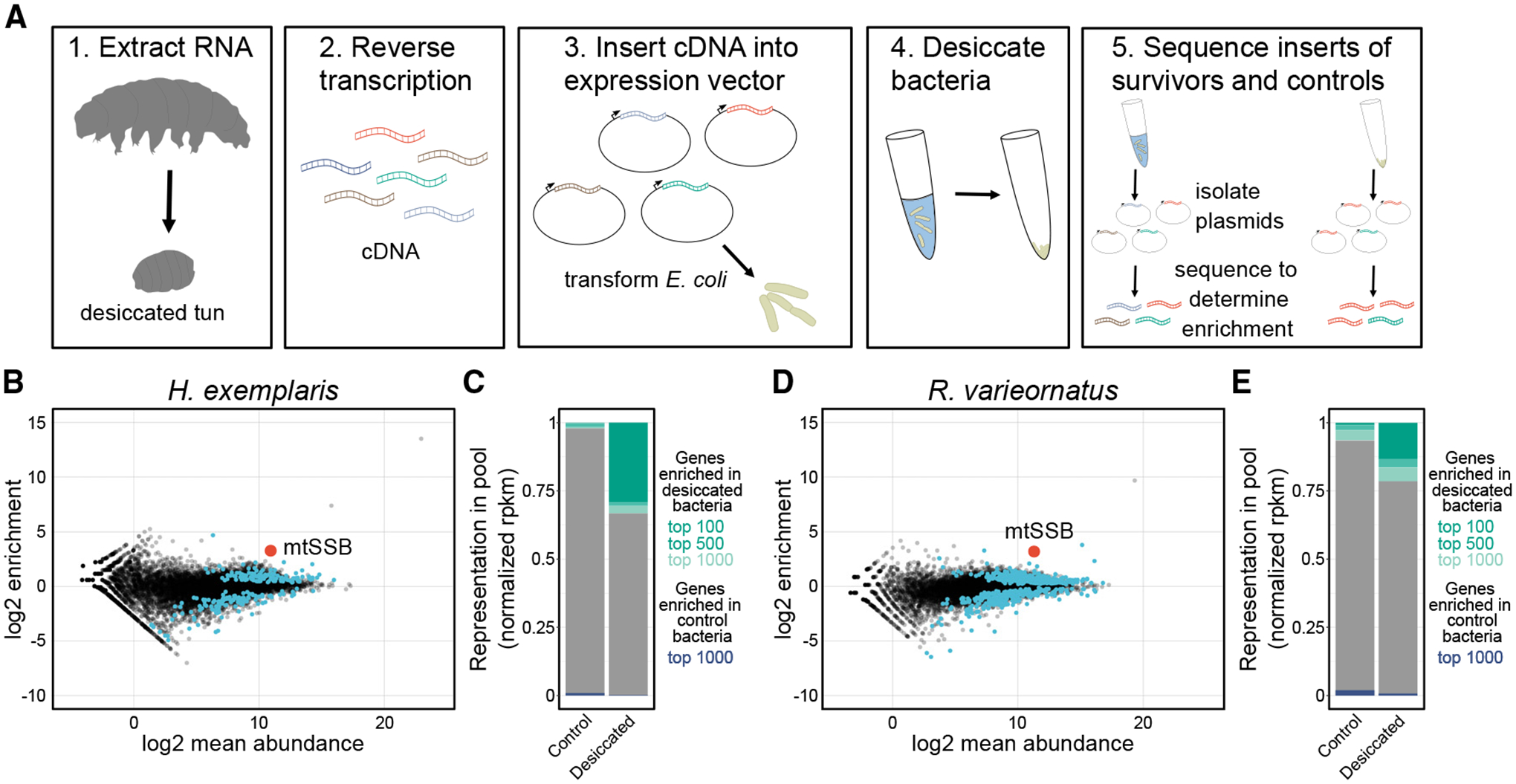
Bacterial expression cloning identified mtSSBs from *R. varieornatus* and *H. exemplaris* as desicco-protectants (A) Schematic of the experimental workflow for expression cloning screens. (B and D) Fold enrichment after desiccation of *H. exemplaris* (B) or *R. varieornatus* (D) genes expressed in bacteria vs. log2 mean abundance (counts per million). mtSSB is highlighted in red. Other genes that were significantly overrepresented in control or desiccated samples are indicated with blue points. (C and E) Stacked bar plots show a representation of the most enriched genes in each library in desiccated and control samples. The top 100, 500, and 1,000 genes that were enriched in desiccated samples are indicated along with the top 1,000 genes that were enriched in control samples. These graphs depict the extent of selection after desiccation.

To identify desicco-protectants from tardigrades, we developed a bacterial expression-cloning approach (Figure 1A). We chose to conduct this screen using *E. coli* because the ability of a eukaryotic protein to function in a prokaryotic cell would suggest the potential for broad functionality that does not rely on eukaryotic binding partners or organelle structures. These types of protectants, which can function in isolation, are most likely to be of use for applications in stabilizing biological materials. Further, *E. coli* can be easily and rapidly grown in large numbers, and there are facile methods for controlled heterologous protein expression. We chose two species of tardigrades as source organisms because they were likely to harbor effective protectants. We selected *Hypsibius exemplaris* (strain Z151) and *Ramazzottius* cf*. varieornatus* (strain YOKOZUNA-1; hereafter referred to as *R. varieornatus* for convenience) as culturable laboratory species with available genomes.^25–27^ Both of these species are eutardigrades of the Hypsibioidea taxon.^28^ *R. varieornatus* exhibits more robust tolerance of desiccation than *H. exemplaris* and does not exhibit as strong transcriptional changes in response to drying.^27,29^ Because some transcripts of *H. exemplaris* are preferentially expressed upon desiccation, we combined RNA isolated from active tardigrades as well as those that had entered the desiccation-tolerant tun state. RNA was reverse transcribed to cDNA and cloned into a bacterial expression vector. The pool of expression plasmids was transformed into *E. coli* BL21-AI. Protein expression was induced in populations of bacteria, and then bacteria expressing tardigrade proteins were split into separate populations and either subjected to desiccation—with generally less than one in a million cells surviving—or maintained as a control group. After rehydration, recovery, and overnight outgrowth of the surviving bacteria, plasmids were isolated, and the cDNA inserts were amplified by linear PCR. We then sequenced the resulting DNA to determine relative enrichment of tardigrade genes in control (non-desiccated) and desiccated samples. 43.9%–51.7% of annotated transcripts from the *H. exemplaris* genome and 51.0%–58.5% of annotated transcripts from the *R. varieornatus* genome were detected in each control library (Figures S1A and S1B), suggesting that we screened approximately 50% of predicted transcripts for each species. For libraries derived from the tardigrade *H. exemplaris*, 118 cDNAs were overrepresented in desiccated samples (compared to controls), and 111 were underrepresented in desiccated samples (compared to controls) (Figures 1B and S1C). The cDNAs enriched in desiccation comprise a substantial portion of the pool that was sequenced, indicative of selection (Figure 1C). For libraries derived from the tardigrade *R. varieornatus*, 385 cDNAs were overrepresented following desiccation, and 284 were underrepresented (Figures 1D, 1E, and S1D).

We found that a cDNA encoding an mtSSB was among the most enriched genes following desiccation for the library derived from *H. exemplaris* and that a cDNA encoding an mtSSB was among the most enriched genes from *R. varieornatus* as well (Figures 1B and 1D). Although the current *H. exemplaris* genome (v.3.1.5) contains sequences of two putative mtSSBs, we confirmed that there is, in fact, one mtSSB (Figures S1G–S1K). Some desicco-protectants are transcriptionally upregulated upon drying, but this does not appear to be the case for *H. exemplaris* mtSSB (Figure S1L).^24,27^ To validate whether mtSSBs are desicco-protectants, we compared their effectiveness in promoting bacterial desiccation survival relative to bacteria carrying an empty vector, expressing GFP, or expressing a truncated form of GFP (tGFP). Expression of tardigrade mtSSBs conveyed a significant increase in desiccation survival relative to these controls (Figure S2A). Each of the proteins expressed was codon optimized for *E. coli*, although we found that codon optimization did not significantly impact mtSSB function (Figure S2B).

**Figure 2.**
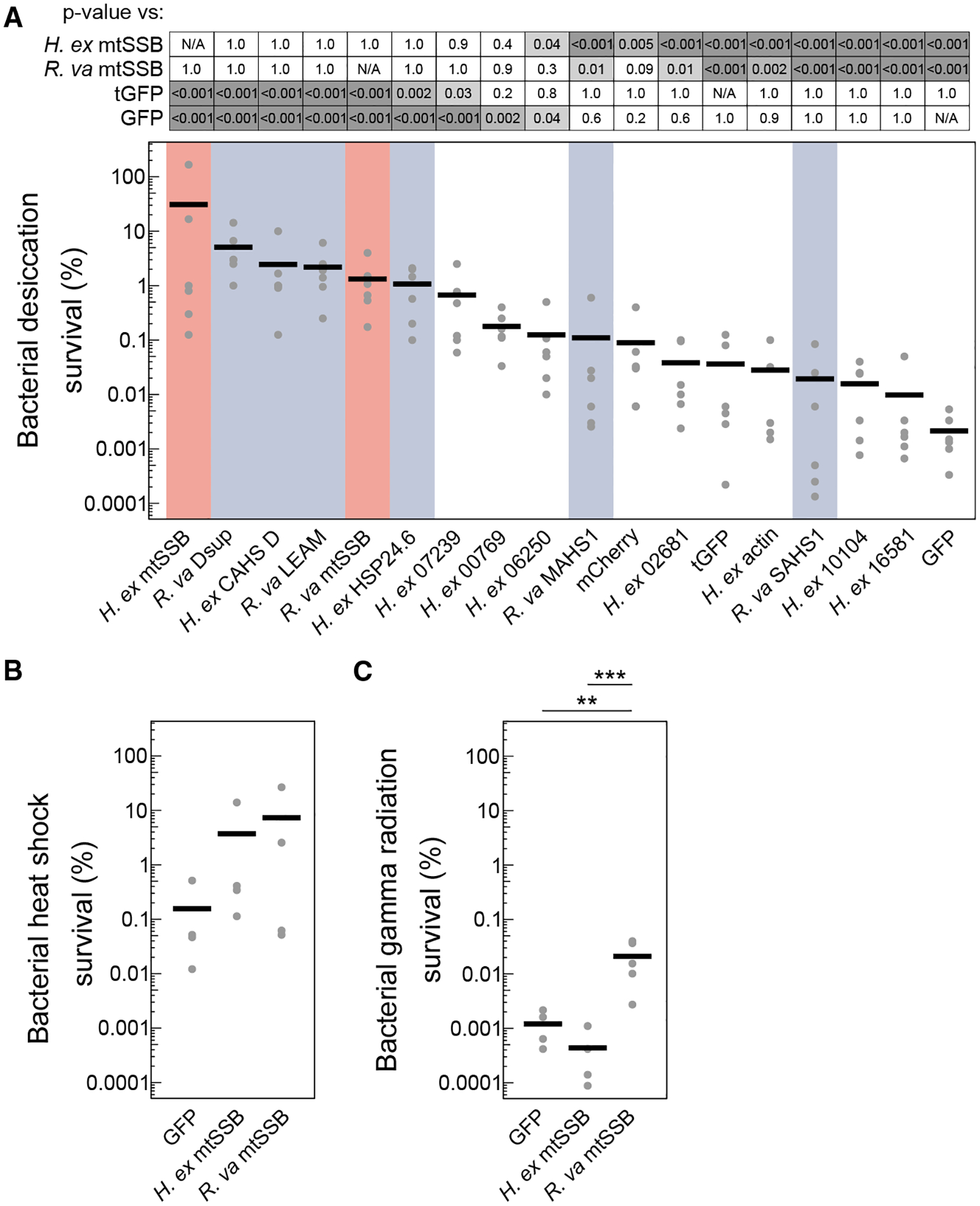
Tardigrade mtSSBs are potent desicco-protectants (A) Desiccation survival of bacteria expressing mtSSBs (red), known protectants (blue), and putative control proteins. There was a significant difference across conditions (*p* < 0.001, 1-way ANOVA, *n* = 6). The *p* values of some pairwise comparisons from a Tukey test are shown (*n* = 6). (B) Survival of bacteria expressing mtSSBs exposed to 52°C heat shock for 1 h. There were no significant differences in survival across conditions (*p* = 0.29, 1-way ANOVA). (C) Survival of bacteria expressing mtSSBs exposed to 2,180 Gy gamma radiation. There was a significant difference in survival across conditions (*p* < 0.001, 1-way ANOVA). Expression of the *R. varieornatus* mtSSB improved survival relative to GFP-expressing controls (*p* = 0.002) and *H. exemplaris* mtSSB (*p* < 0.001, Tukey test). Individual data points in each graph represent independent replicates, and bars indicate mean values. **p < 0.01, ***p < 0.001.

To determine whether mtSSBs harbor potency similar to known desicco-protectants, we compared survival of bacteria expressing mtSSBs to that of cells expressing some of the most effective tardigrade protectants reported in the literature to date, including CAHS D, Dsup, a mitochondrial late embryogenesis abundant protein, and a small heat shock protein, HSP24.6^22–24,30,^.^31^ These known protectants likely function by a variety of unique mechanisms, including cellular vitrification, stabilizing DNA, protecting membranes, and limiting protein aggregation.^23,24,30,32–38^ We also included a number of additional putative controls, including transcripts that were downregulated in dried tardigrades (Figure S2C), actin, and fluorescent proteins, which further served as a visual control for protein expression (Figure S2D). Expression of each tardigrade mtSSB improved desiccation survival over 100-fold compared to several control proteins and to levels that were statistically indistinguishable from the strongest known tardigrade desicco-protectants (Figure 2A). We conclude that both species of tardigrades harbor mtSSBs that can potently extend the tolerance of bacterial cells to desiccation.

It is possible that mtSSBs could improve bacterial desiccation survival via indirect mechanisms. We wanted to test whether such changes or other idiosyncrasies of heterologous expression could explain the difference in desiccation survival of mtSSB-expressing cells. To do so, we measured levels of protein expression and solubility as well as the growth rate of cells. Although there was variability in expression levels and solubility of proteins (Figure S2E), these factors did not explain the efficacy of mtSSBs: there was no correlation between these variables and desiccation survival (Figure S2F). Similarly, expression of different proteins led to differential effects on bacterial growth, but this did not correlate strongly with desiccation survival either (Figures S2G and S2H).

Another possible explanation for the efficacy of heterologously expressing mtSSBs is that they could activate endogenous bacterial stress response pathways, such as the SOS response, which is induced to cope with DNA damage.^39–41^ Overexpression of *E. coli* SSB and human SSBP1 has been shown to induce modest amounts of DNA damage.^42^ It is conceivable that heterologous expression of tardigrade mtSSBs may cause similar DNA damage and trigger the SOS response. Upon damage to DNA and recognition of exposed single-stranded DNA (ssDNA) during replication by *RecA*/RAD51, the transcriptional repressor *lexA* is cleaved, leading to the de-repression of more than 40 genes.^43–48^ The accumulation of these gene products contributes to DNA damage repair, error-prone DNA synthesis, and filamentous growth.^44^ Filamentous growth is a conspicuous cellular phenotype that results from the de-repression of *sulA*, leading to inhibition of *ftsZ* and cell division.^49,50^ We measured filamentous growth in mtSSB-expressing *E. coli* to determine whether activation of the SOS response might explain their desiccation-protective effect. Expression of the *H. exemplaris* mtSSB caused an increase in bacterial length, but expression of the full-length version of the *R. varieornatus* protein did not induce filamentous growth in bacteria, suggesting that its survival benefit in these cells is not primarily due to activation of the SOS response (Figures S2I and S2J). To determine whether SOS response genes may nonetheless be activated in *R. varieornatus* mtSSB-expressing cells, we conducted mRNA sequencing to search for a transcriptional signature of SOS activation. There was not a coherent upregulation of *lexA*-regulated transcripts, suggesting that the canonical de-repression associated with SOS was not present in these bacteria (Figure S2K).^46,51^ Therefore, the potency of *R. varieornatus* mtSSB in improving bacterial desiccation survival is likely not due to activation of the SOS response.

Some of the types of cellular damage incurred during desiccation overlap with the types of damage that result from other stresses. For example, proteins are prone to aggregation during desiccation or at high temperature, and DNA is prone to damage under desiccation or ionizing radiation.^30,52–56^ We were curious whether mtSSBs might provide protection against other stresses, like the proteotoxicity of heat stress or DNA damage of ionizing radiation. While mtSSBs could significantly improve bacterial desiccation survival, they did not have a significant effect on survival of heat stress (Figure 2B). However, the expression of *R. varieornatus* mtSSB did convey a modest benefit in protecting cells against ionizing radiation (Figure 2C). We conclude that mtSSBs are potent desicco-protectants and may provide some cross-tolerance to other DNA-damage inducing stresses.

### mtSSBs promote desiccation tolerance by binding to DNA

**Figure 3.**
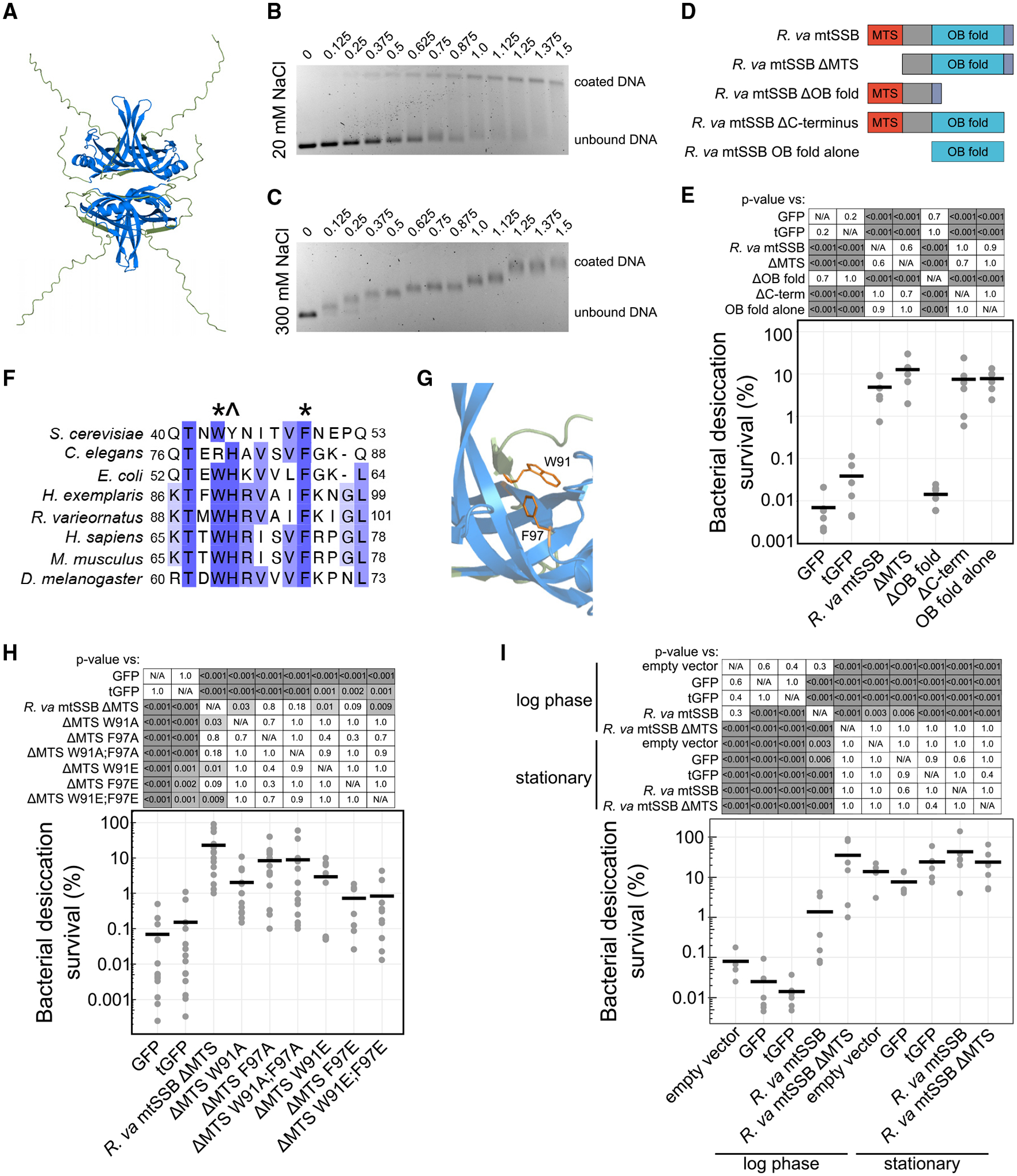
mtSSBs protect against desiccation by binding DNA (A) The predicted tetrameric structure of *R. varieornatus* mtSSB ΔMTS. The highly conserved oligonucleotide/oligosaccharide binding (OB) fold is colored blue. (B) Gel-shift analysis of *R. varieornatus* mtSSB ΔMTS at low salt concentration (20 mM NaCl) reveals cooperative binding to ssDNA (M13mp18 circular DNA). (C) Gel shifts conducted in buffer with 300 mM NaCl reveal incremental, non-cooperative binding of the protein to ssDNA. (B and C) Numbers above each lane of the gels indicate the ratio of protein:DNA. (D) A schematic of domain deletion constructs of *R. varieornatus* mtSSB. (E) Desiccation survival of bacteria expressing *R. varieornatus* mtSSB constructs. The OB fold is necessary and sufficient for bacterial desiccation survival (*n* = 6). (F) A portion of sequence alignment of mtSSBs with conserved tryptophan (W) and phenylalanine (F) residues indicated with asterisks. These residues are likely important for the DNA-binding ability of the protein. The conserved histidine (H), annotated with ^, is important for dimerization of the protein. (G) The tryptophan and phenylalanine side chains extend into the DNA-binding pocket of the OB fold and are important for stacking interactions when binding nucleotides. (H) Desiccation survival of bacteria expressing *R. varieornatus* mtSSB ΔMTS with point mutations that convert the aromatic W or F residues to either alanine (A) or glutamic acid (E) (*n* = 7–14). (I) Bacteria in stationary phase were significantly more tolerant of desiccation than those in log phase. The desicco-protective effects of mtSSB expression were specific to replicating cells (*n* = 6). (E, H, and I) The *p* values were calculated with Tukey tests following significant 1-way ANOVA results.

To determine the mechanism by which tardigrade mtSSBs can promote desiccation tolerance, we focused our attention on *R. varieornatus* mtSSB because it likely does not induce the SOS response (Figures S2I–S2K). The predicted tetrameric structure of *R. varieornatus* mtSSB is similar to that of other mtSSBs as well as prokaryotic SSBs with core oligonucleotide/oligosaccharide binding (OB) folds (Figure 3A).^57–59^ Notably, both *H. exemplaris* and *R. varieornatus* mtSSBs lack a disordered linker region and acidic C-terminal tip that are characteristic of the endogenous *E. coli* SSB.^60^ Instead, tardigrade mtSSBs have a longer disordered N-terminal tail. Because the acidic tip of the *E. coli* SSB is important for recruiting binding partners,^61^ we did not think it likely that heterologous expression of mtSSBs would improve desiccation survival by recruiting endogenous bacterial DNA repair machinery. However, the predicted structural conservation of *R. varieornatus* mtSSB suggested its ability to bind DNA. To directly test for DNA binding, we purified *R. varieornatus* mtSSB without the predicted mitochondrial targeting sequence (ΔMTS). We assessed DNA binding by gel-shift assays, combining protein and M13mp18 ssDNA at defined ratios.^62,63^ Purified *R. varieornatus* mtSSB ΔMTS demonstrated cooperative DNA binding at a low salt concentration (20 mM NaCl), as indicated by bimodal bands in the gel (Figure 3B). At a higher salt concentration (300 mM NaCl), binding to DNA was non-cooperative, instead showing incremental increases in the location of bands with increasing protein concentrations (Figure 3C). These salt-dependent binding modes of *R. varieornatus* mtSSB mimic those of the *E. coli* SSB—evidence for the general conservation of the DNA-binding function of these proteins and ability to engage in both cooperative and non-cooperative interactions with ssDNA.^62–64^

To determine whether DNA binding was essential for the desicco-protective function of mtSSBs, we tested the ability of constructs containing different regions of the protein to protect desiccated cells (Figures 3D and 3E). The MTS and C-terminal region were dispensable for protection (Figures 3E, S3A, and S3B). The OB fold was necessary and sufficient to strongly improve survival of desiccated bacteria (Figure 3E). Notably, bacteria expressing *R. varieornatus* mtSSB ΔMTS exhibited more filamentous growth than controls (Figure S3B), which may contribute in part to enhanced desiccation tolerance of this strain. However, expression of the OB fold alone improved bacterial desiccation survival without impacting bacterial length (Figures 3E and S3B). Therefore, it is unlikely that SOS activation and elongation explain the desicco-protective properties of this construct. Collectively, these data reveal that DNA binding is likely the salient functional property for improving desiccation survival.

We leveraged previous work that has been carried out on the mechanisms of SSB-DNA binding to specifically test the hypothesis that DNA binding is essential for desicco-protection. Protein sequence alignments revealed conservation of a tryptophan (W) and phenylalanine (F) that have been shown to contribute to DNA binding (Figure 3F; supplemental alignment 1).^65^ These aromatic amino acids extend into the ssDNA-binding pocket of the OB fold and stabilize ssDNA by binding with base-stacking interactions (Figure 3G).^57^ Mutations in these residues reduced the protective ability of the proteins, with a substitution to negatively charged glutamic acid (E) at either site generally providing a more significant effect than substitution with likely less disruptive alanine (A) (Figures 3H, S3C, and S3D). These data lend further support for the conclusion that DNA binding is essential for the desicco-protective ability of mtSSB.

ssDNA is exposed by normal cellular functions like replication and transcription as well as during genotoxic stress.^66^ Given the exposure of ssDNA during DNA replication and our results implicating SSBs as strong protectants, we predicted that the protective effects of mtSSB might be cell cycle dependent. To test this hypothesis, we assessed the protective capacity of mtSSB in log- and stationary-phase cells. Cells in stationary phase were generally more tolerant of desiccation, as has been observed in bacteria and yeast.^67–70^ Cells in stationary phase are also more tolerant of other stresses, such as temperature and oxidative stress, at least in part due to upregulation of protectants like trehalose, which may confer protection during desiccation as well.^71,72^ As we consistently observed, expression of *R. varieornatus* mtSSB (with or without MTS) in replicating cells improved desiccation survival (Figures 2A, 3E, and 3I); however, we found that there was not a significant effect of mtSSB overexpression in stationary-phase cells (Figures 3I and S3E). This could indicate that exposed ssDNA at replication forks may be vulnerable to damage in the face of desiccation or that other protective mechanisms of stationary-phase cells may mask the added benefit of mtSSB expression. We conclude that the vulnerability of log-phase cells to desiccation can be potently ameliorated by mtSSB binding to DNA.

### Tardigrade mtSSBs can localize to mitochondrial DNA and provide a modest increase in yeast desiccation survival

In bacteria, tardigrade mitochondrial SSBs may function similarly to endogenous SSBs, with access to genomic DNA. However, in eukaryotic cells, mtSSBs localize to mitochondria, and the replication protein A (RPA) complex functions analogously in the nucleus. The tardigrade mtSSBs we identified have predicted MTSs. To determine whether tardigrade mtSSBs can localize to mitochondria, we added GFP tags for visualization and expressed the proteins in *S. cerevisiae* (Figure S4A). The full-length versions of both *R. varieornatus* and *H. exemplaris* mtSSBs exhibited punctate localization that overlapped with an endogenous mitochondrial Cox4::mCherry tag (Figure S4B). Expression of the MTS from each protein fused to GFP resulted in more diffuse localization throughout mitochondria. Deleting the MTS from each protein resulted in cytosolic localization without discernable puncta. We confirmed these localization patterns in yeast stained with MitoTracker (Figure S4C). We hypothesized that the punctate distribution of mtSSBs within the mitochondria could represent binding to mtDNA. Indeed, in cells stained with DAPI, mtSSB puncta overlapped with DAPI-labeled mtDNA (Figure S4D). Thus, tardigrade mtSSBs expressed in yeast likely associate with mtDNA.

While mtSSBs were highly effective in protecting desiccating bacteria, it was unclear whether they would demonstrate the same potency in a eukaryotic cell—with its diverse set of organelles that could be harmed by desiccation—when localized only to mitochondria. We compared desiccation survival of yeast expressing tardigrade mtSSBs in different compartments to determine whether localization of mtSSBs in mitochondria could also serve a protective role. Yeast expressing *H. exemplaris* mtSSB that localized to mitochondria had slightly improved desiccation survival compared to yeast expressing the cytosol-localized version of the same protein (Figure S4E). This suggests that this mtSSB can confer at least some protection when localized to mitochondria.

### The ability to protect *E. coli* from desiccation is a shared property of the ssDNA-binding domain of some SSBs

**Figure 4.**
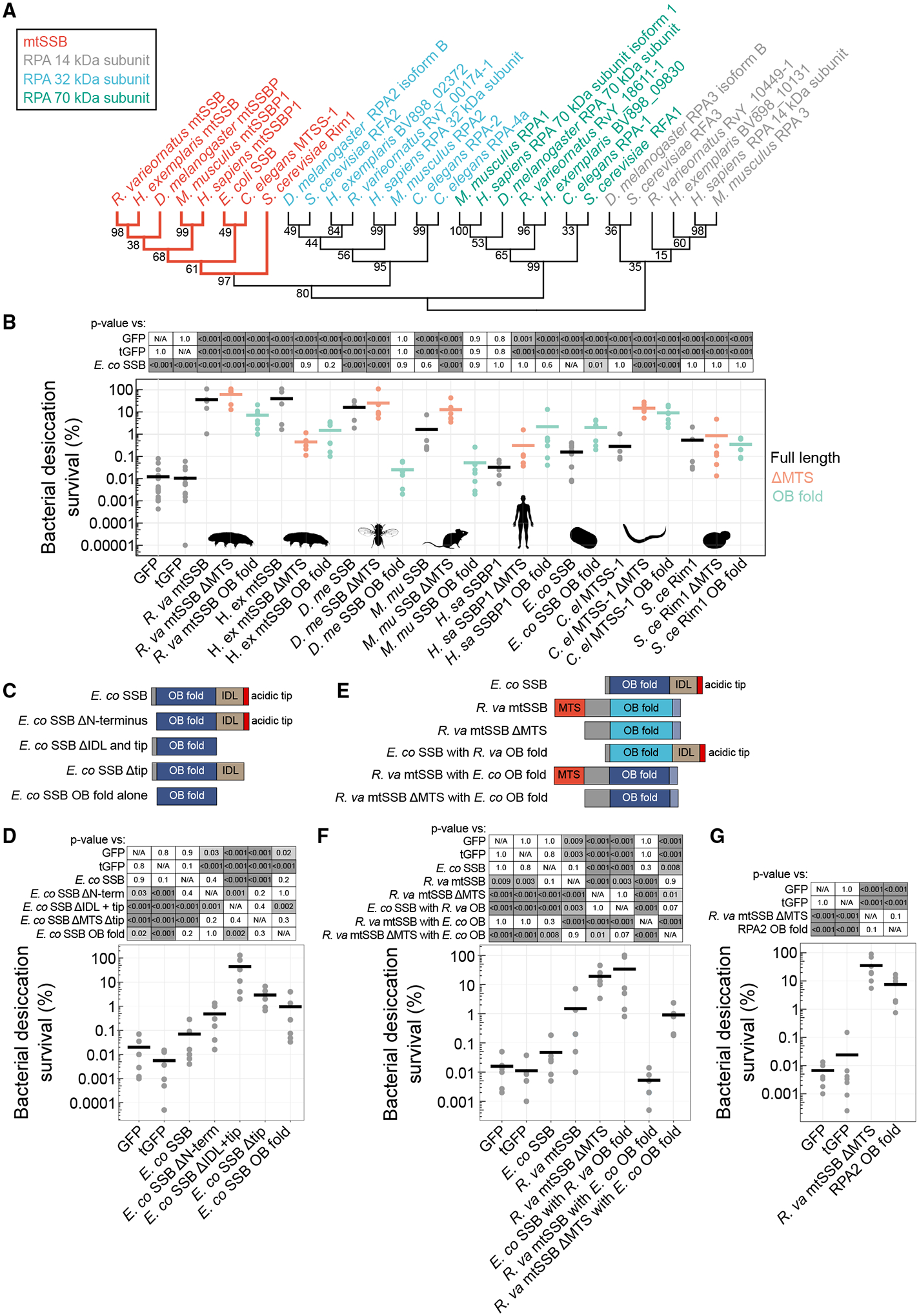
Multiple proteins with OB fold domains can provide desicco-protection to *E. coli* (A) A maximum-likelihood gene tree depicts relationships between protein sequences of homologs of RPA subunits and mtSSBs from multiple species. Numbers at branchpoints indicate bootstrap values. (B) Desiccation survival of bacteria expressing full-length mtSSBs from different species, mtSSBs without mitochondrial targeting sequences (MTSs), or expressing exclusively the OB folds of these proteins (*n* = 5–11). (C) A schematic of domain deletion constructs of *R. varieornatus* mtSSB. (D) Desiccation survival of *E. coli* expressing each of the constructs depicted in (C) (*n* = 6). (E) A schematic of chimeric constructs interchanging OB folds between the *E. coli* SSB and *R. varieornatus* mtSSB. (F) Desiccation survival of *E. coli* expressing each chimeric construct as shown in (E) (*n* = 6). (G) Expression of the consensus OB fold from RPA2 (NCBI conserved protein domain family cd03524) was sufficient to improve bacterial desiccation tolerance (*n* = 7). (B, D, F, and G) The *p* values were calculated with Tukey tests following significant 1-way ANOVA results.

Our finding that tardigrade mtSSBs’ ssDNA-binding activity makes them potent desicco-protectants suggested to us that coating ssDNA with proteins can contribute to protecting desiccating cells and, hence, that mtSSBs from other organisms would share this potency. mtSSBs are widely conserved proteins (Figure 4A). Eukaryotes have both mitochondrial and nuclear SSBs; typically, one copy of an mtSSB and one copy each of three nuclear RPA subunits (RPA1, 70 kDa; RPA2, 32 kDa; and RPA3, 14 kDa). One exception is *C. elegans*, which does not have a homolog of the RPA3 subunit but instead has another RPA2 paralog, RPA-4.^73^ Expression of mtSSB proteins (either full length or ΔMTS) from tardigrades (*R. varieornatus* and *H. exemplaris*), fruit flies (*D. melanogaster*), nematodes (*C. elegans*), mouse (*M. musculus*), and yeast (*S. cerevisiae*) was indeed sufficient to increase bacterial desiccation survival (Figures 4B and S5A–S5D). However, the overexpression of *H. exemplaris* or *D. melanogaster* full-length mtSSBs caused filamentous growth, suggesting that survival benefits may be indirect (Figure S5B). Tardigrade mtSSBs were among the most potent of those tested. Human mtSSB1 was only sufficient to improve desiccation tolerance when lacking its MTS (Figure 4B). In general, full-length and ΔMTS proteins affected bacterial desiccation survival similarly, although some differences, like with *C. elegans* MTSS-1 (full length vs. ΔMTS, *p* < 0.001, Tukey test) and *H. exemplaris* mtSSB (full length vs. ΔMTS, *p* = 0.01, Tukey test), could reflect differences in protein folding, stability, or DNA binding affinity in a cleaved vs. uncleaved state.^74^ Although the MTSs are predicted to be cleaved, we do not yet know whether this happens *in vivo* in tardigrades.

The OB fold in isolation from many mtSSBs could also improve desiccation survival (Figures 4B, S5E, and S5F). This finding suggests that, like with the *R. varieornatus* mtSSB (Figure 3E), DNA-binding activity of mtSSBs is likely sufficient to explain their protective function. Interestingly, overexpression of the endogenous *E. coli* SSB had a significantly weaker effect than many of the proteins from other organisms (Figure 4B) despite strong expression and solubility (Figure S5A). It is possible that the effect of *E. coli* SSB is already saturated by endogenous expression of the protein or that overexpression could lead to dysregulation of SSB binding partners that contribute to essential processes like replication and the DNA damage response. Specifically, overexpression of *E. coli* SSB may titrate away binding partners required for normal cellular functions. To test this hypothesis, we expressed heterologous constructs with deletions of various domains of the *E. coli* SSB, including the acidic C-terminal tip, which is essential for interactions with binding partners (Figure 4C).^61,75–77^ Deletion of the acidic tip or the acidic tip and the intrinsically disordered linker (IDL) improved the efficacy of the protein compared to the full *E. coli* SSB (Figures 4D, S6A, and S6B). The improved function of the truncated protein, which is likely deficient for interacting with binding partners, suggests that DNA binding but not recruitment of repair proteins explains the means of protection. Similarly, the non-native mtSSB proteins and the OB folds of those proteins lack the binding sites to recruit *E. coli* SSB binding partners. Because these proteins still protected against the lethality of desiccation, we conclude that DNA binding of these proteins can improve desiccation tolerance without recruitment of functional binding partners.

We were further interested in whether sequence differences in OB folds could contribute to differences in desiccation tolerance between the *E. coli* and *R. varieornatus* proteins. We constructed chimeric variants, swapping the OB folds between the *E. coli* SSB and *R. varieornatus* mtSSB (Figure 4E). Interestingly, adding the *E. coli* SSB to the *R. varieornatus* mtSSB generally reduced the protein’s desicco-protective effect (Figure 4F). Conversely, adding the *R. varieornatus* mtSSB OB fold to the *E. coli* SSB improved its efficacy (Figure 4F). However, the inclusion of the *R. varieornatus* OB fold in the *E. coli* protein led to increased bacterial length, so the effect on desiccation survival may be indirect (Figures S6C and S6D). It is also possible that the inclusion of the *R. varieornatus* mtSSB OB fold may disrupt interactions with endogenous binding partners, similar to deletion of the C-terminal regions of the *E. coli* SSB. This, too, could contribute to increased desiccation tolerance. Although chimeric variants likely alter multiple aspects of the folding and function of the proteins, it is nonetheless interesting to speculate that intrinsic differences in OB fold domains could be exploited to engineer a sequence optimized for binding DNA to protect drying cells.

We next inquired whether eukaryotic nuclear SSBs (RPAs) could promote desiccation tolerance. RPAs assemble as heterotrimers and carry out functions similar to mtSSBs. They also contain OB fold domains that facilitate DNA binding. None of the RPA subunits from either *H. exemplaris* or *R. varieornatus* were significantly enriched in our screen, perhaps due to limited potency of individual subunits without assembly of the heterotrimeric complex or the fact that mitochondrial proteins like mtSSB may already be poised to function in bacteria. To determine whether the nuclear analog of mtSSBs harbors similar potency as desiccation protectants, we measured desiccation survival of bacteria expressing a consensus OB fold from the RPA2 family (consensus protein sequence from NCBI: cd03524). The generalized RPA2 OB fold was sufficient to improve desiccation survival to a level that was indistinguishable from *R. varieornatus* mtSSB ΔMTS (Figures 4G, S6E, and S6F). We conclude that diverse OB fold-containing ssDNA-binding proteins can protect bacteria under desiccation. Our findings support the model that coating ssDNA with proteins can contribute to protecting desiccating cells.

## DISCUSSION

We set out to screen for further protectants in a way that enriches for selection of protectants that could function in heterologous contexts and would not rely on criteria like endogenous transcriptional upregulation, shared biochemical properties with known protectants, or sequence similarity to known protectants. Our bacterial expression cloning screens identified tardigrade mtSSBs as conserved proteins that dramatically improved bacterial desiccation survival. Indeed, this approach revealed a potency of mtSSBs as desicco-protectants even though they are not transcriptionally upregulated in *H. exemplaris* during desiccation (Figure S1L).^24,27^ The lack of transcriptional induction of mtSSBs during desiccation in tardigrades may reflect either a sufficient constitutive presence of the protein or potentially a lack of involvement *in vivo* in the desiccation response.^24,27,78^ It is also likely that mtSSB regulation specifically in response to drying is constrained by other factors because mtSSBs play essential functions in regulating mitochondrial DNA replication and physiology.^79–81^ Therefore, a strength of our expression-cloning approach is its effectiveness in identifying functional properties of proteins while remaining agnostic to the functions of those proteins in the source organism. In this way, screens employing expression cloning coupled with genetic selection, like the one we developed, present an efficient method to identify functional properties of proteins even from emerging model organisms that are not amenable to traditional forward genetic screening. It also allows the discovery of potent protectants that do not rely on their native environments and binding partners to function.

Given the endosymbiotic origin of mitochondria, it is perhaps unsurprising that our approach identified a mitochondrial protein that could function well in a prokaryote.^82^ While mtSSBs were strong desicco-protectants in bacteria, in yeast this effect was muted (Figure S4E). It is possible that high copy numbers of mitochondrial genomes could make mtDNA protection less essential or that the protective effect may be more apparent when mtSSBs act in concert with other protectants functioning in other organelles.^83^ In general, the increased complexity of eukaryotic cells, with many subcellular compartments that could be harmed by desiccation, will likely limit the potency of some protectants that work well individually in prokaryotes. Expression cloning screens carried out in eukaryotic cells may reveal more protectants and perhaps some that can work in the context of different subcellular compartments to protect cells from extreme stress.

We demonstrate that DNA binding is essential for mtSSBs to confer desicco-protection (Figure 3). Because *R. varieornatus* mtSSB exhibited non-cooperative binding at higher salt concentrations (Figure 3C), we predict that non-cooperative binding is likely the predominant mode, as osmolytes become concentrated when drying cells lose water.^3^ In support of this prediction, the *E. coli* SSB lacking the IDL and acidic tip, which collectively contribute to cooperativity, was still able to promote desiccation tolerance—doing so more effectively than the full length *E. coli* SSB (Figure 4D).^76^ While the ability to bind DNA is necessary and sufficient for mtSSBs to promote desiccation tolerance, there are likely other properties of the proteins that can contribute as well. *S. cerevisiae* Rim1 has weaker dimerization affinity than other mtSSBs due to the presence of a tyrosine instead of a highly conserved histidine at the dimer interface (Figure 3F).^84,85^ This provided the specific hypothesis that the reduced efficacy of Rim1 to promote bacterial desiccation tolerance relative to *R. varieornatus* mtSSB (Figure 4B) may be due to reduced dimerization affinity. Indeed, a version of the *R. varieornatus* mtSSB carrying an H92Y mutation had reduced desiccation survival relative to the wild-type protein (Figures S7A–S7C). Other amino acid substitutions to replace H92 of *R. varieornatus* mtSSB produced similar effects (Figures S7D–S7F). Thus, dimerization affinity may impact the level of desicco-protection conferred by mtSSBs, perhaps through altered DNA binding affinity or other functions dependent on tetramer assembly.^84^ Other properties of SSBs could feasibly contribute to desiccation tolerance as well, like phase separation or effects on RNA.^86–88^

SSBs are non-specific in binding ssDNA.^64^ Therefore, we envision a mechanism similar to what has been proposed for Dsup: protein can coat DNA to limit damage during genotoxic stresses, including desiccation.^23,32–34^ Although *in vivo* roles for Dsup have not been demonstrated, the potency of Dsup has been demonstrated in a number of contexts, but it has also been reported to alter normal transcriptional regulation and may itself promote DNA damage in some contexts.^32,89,90^ Similarly, we observed that overexpression of *H. exemplaris* mtSSB in *E. coli* induced filamentous growth characteristic of the SOS DNA damage response (Figures S2I and S2J). This suggests that the presence of DNA-coating proteins like Dsup or mtSSBs may be advantageous during extreme stress but may prove disruptive under normal physiological conditions. Thus, these proteins might be thought of as double-edged swords able to confer protection in some contexts but possibly inducing damage or dysregulation in others. mtSSBs differ from Dsup in their affinity for ssDNA versus a preference for nucleosome-bound double-stranded DNA (dsDNA).^34^ ssDNA is generally prone to damage, and ssDNA breaks are a prominent form of DNA damage in tardigrades during short term desiccation, while dsDNA breaks occur over longer timescales.^54,55,91^ Although we have shown that *R. varieornatus* mtSSB binds to ssDNA, it is possible that, at high concentrations during heterologous expression, these proteins may also associate with dsDNA.

We found that the potency of mtSSBs in bacteria is specific to replicating cells, which are especially vulnerable to desiccation damage, possibly due to exposed ssDNA at active replication forks. DNA wrapped around mtSSBs might be physically buffered against DNA damage and ensuing lethality. It is conceivable that such protective mechanisms could be found in either mitochondria or nuclei of eukaryotic cells to promote desiccation tolerance. Such protection might offer advantages in contexts beyond just desiccation. Better understanding mechanisms by which ssDNA-binding activity can act as a potent contributor to DNA stability and stress tolerance will be of interest.

### Limitations of the study

This study utilized expression cloning as a method to identify proteins that can serve as protectants in heterologous contexts. This approach effectively identified mtSSBs as potent desicco-protectants. However, there are likely many other proteins that could serve as desicco-protectants that were not captured by this approach. For example, truncated cDNAs, cDNAs cloned out of frame, or proteins that require other binding partners would not surface. Thus, there may well be many other desicco-protectants that remain to be discovered.

We chose to employ an approach that prioritized protectants that can work beyond their native context of a tardigrade in order to identify proteins with greater potential for broad applicability. This approach is inherently constrained by the biology of the host cell and, notably, does not address another important question: which protectants function endogenously to promote desiccation tolerance of tardigrades? The discovery of a desicco-protective role for a protein that is well-conserved, even among desiccation-sensitive organisms, suggests that our screening approach may have been successful even with RNA derived from non-anhydrobiotic organisms. Other approaches will be required to determine mechanisms of protection *in vivo* and to identify protectants that function endogenously in anhydrobiotes.

## RESOURCE AVAILABILITY

### Lead contact

Requests for further information and resources should be directed to and will be fulfilled by the lead contact, Jonathan D. Hibshman (jhibshman@smu.edu).

### Materials availability

Plasmids for protein expression in bacteria and yeast generated in this study are available upon request.

### Data and code availability

- All data are available in Data S1. Raw sequencing data are available through the NCBI Sequence Read Archive (SRA): BioProject PRJNA1008366 and PRJNA1070671.
- This paper does not report original code.
- Any additional information required to reanalyze the data reported in this paper is available from the lead contact upon request.

## STAR★METHODS

### EXPERIMENTAL MODEL AND STUDY PARTICIPANT DETAILS

#### Hypsibius exemplaris

*Hypsibius exemplaris* (strain Z151) cultures were maintained in vented 35 mm petri dishes (Tritech Research, T3500) with spring water (Deer Park) and were fed *Chlorococcum* sp. (Carolina Biological, Item #152091). Cultures were kept at room temperature (~22°C) and animals were moved to fresh cultures approximately every two weeks with an aspirator tube assembly (Millipore Sigma, A5177) and a needle pulled from a glass capillary tube (World Precision Instruments, 1B100F-4). All tardigrades were parthenogenetic (female).

#### Ramazzottius varieornatus

Cultures of parthenogenetic (female) *Ramazzottius* cf. *varieornatus* (YOKOZUNA-1) were similarly maintained in vented 35 mm petri dishes at room temperature (~22°C) and moved to fresh dishes approximately every two weeks. *R. varieornatus* were fed SuperFresh Chlorella V12 (Reed Mariculture).

### METHOD DETAILS

#### Expression library construction

To create bacterial expression libraries, we first extracted RNA from *Hypsibius exemplaris* (strain Z151) and *Ramazzottius* cf. *varieornatus* (strain YOKOZUNA-1). For *H. exemplaris*, RNA was isolated from both active adult tardigrades and some that were in the tun state. To induce tun formation, tardigrades were transferred to plastic petri dishes coated with a thin film of 2% agar and conditioned at 97.5% relative humidity in a desiccation chamber as previously described.^92,94^ For *R. varieornatus*, which does not exhibit a significant transcriptional response to desiccation,^27^ total RNA was isolated only from active adult animals. Total RNA was extracted from several hundred tardigrades using a PicoPure RNA isolation kit (Applied Biosystems, Cat #KIT0204). A Cloneminer II cDNA Library Construction Kit (Invitrogen, Cat #A11180) was used to generate cDNA with oligo(dT) priming, and clone it into plasmids for bacterial expression according to the specifications in the manual. Briefly, the first strand of cDNA was synthesized from total RNA by reverse transcription (SuperScript III RT), the second strand was synthesized with *E. coli* DNA Polymerase I, and T4 DNA Polymerase created blunt-ended cDNA. attB adapters were ligated onto cDNA with T4 DNA ligase. Adapter-ligated cDNAs were fractionated on 1 mL Sephacryl S-500 HR resin columns to remove residual non-ligated adapters. Adapter-ligated cDNAs were then cloned into pDONR222 with BP clonase II. The entry libraries contained an estimated 4.6 million clones (*H. exemplaris*) and 2.7 million clones (*R. varieornatus*).

The donor vectors carrying tardigrade cDNA inserts were transformed into ElectroMAX DH10B T1 Phage Resistant Cells using an Electroporator 2510 (eppendorf) set to a voltage of 2.2 kV. After 1 h of recovery, transformants were frozen at −80°C. To isolate donor plasmids, cells were thawed and grown up in 50 mL LB with Kanamycin selection (50 μg/mL, Sigma, K-1377). Plasmids were harvested with a HiPure Plasmid Filter Midiprep Kit (Invitrogen, Cat #K210014). The library transfer reaction with LR Clonase II was carried out to move inserts into pDEST17 (Invitrogen, Cat #11803012) to generate an expression library. The expression libraries contained an estimated 1.7 million clones (*H. exemplaris*) and 25.7 million clones (*R. varieornatus*).

#### Screening expression libraries

Expression libraries were transformed into *E. coli* BL21-AI (Invitrogen, Cat #C607003). 150 ng of plasmid DNA was transformed into a total of 300 μL of competent cells for expression screens. Two independent library transformations were conducted. In each case, after initial recovery for 30 min in Super Optimal broth with Catabolite repression (S.O.C.) media (Invitrogen, Cat #15544034), transformed pools of bacteria were grown overnight in Luria-Bertani (LB) liquid media (10g tryptone, 10g NaCl, 5g yeast extract, H_2_O to 1L) with 100 μg/mL ampicillin (Sigma, A0166). Cultures were diluted 1:20 into LB with ampicillin and 0.2% L-arabinose to induce protein expression and split into different cultures at this stage for technical replicates. After 4 h of induction, for each technical replicate 3 mL of culture was collected for a control sample and 3 mL of culture was collected for desiccation. Plasmids were isolated from controls with a Purelink HQ Mini Plasmid Purification Kit (Invitrogen, Cat #K2100-01). Bacteria that were to be desiccated were concentrated by centrifugation (5,000 rpm, 10 min), washed with 0.85% NaCl, and retained as a pellet. Tubes were desiccated overnight in a Savant speedvac concentrator and then kept for four days in a desiccation chamber with drierite desiccant. After desiccation, bacteria were rehydrated and allowed overnight outgrowth of survivors in LB with ampicillin. As with controls, plasmids were isolated from these populations of desiccation survivors with minipreps (Invitrogen, Cat #K2100-01).

#### Sequencing

To capture biological and technical variability in this screen, we sequenced two technical replicates (i.e., two controls and the paired desiccated samples) from each of the two independent library transformations. To avoid sequencing primarily pDEST17 plasmid backbone, the cDNA inserts of the plasmids were isolated and amplified by low-cycle (12x) PCR using a Kapa2G Robust Hotstart PCR kit (Roche, Ref #07961073001) according to specifications. Primer sequences were CATCACCATCACCTCGAATCAAC and TTCGGGCTTTGTTAGCAGCCTCGAATC. The annealing temperature for the reaction was 55°C and the extension time was 2 min. The protocol for the kit suggests 15 s/kb, thus allowing for amplification of inserts up to ~8 kb. These PCR amplicons were cleaned with a ChargeSwitch-Pro PCR cleanup kit (Invitrogen, Cat #CS32050). Amplicons were sonicated to ~200 bp and sequencing libraries were prepared by the UNC high-throughput sequencing facility (HTSF) using the Kapa DNA hyper kit. Batches were pooled onto lanes for paired end 75 bp read sequencing on a HiSeq4000.

#### Sequence analysis

Quality of reads from sequencing was assessed with fastqc. Paired reads were mapped to the genomes of *H. exemplaris* (v3.1.5) or *R. varieornatus* (Rv1) with bowtie2.^27^ Mapped reads were assigned to genes with featurecounts. Genes without any reads mapped to them were removed prior to determining enrichment in control or desiccated conditions with DESeq2.^93^ Genes with an FDR <0.05 were considered to be significantly enriched in desiccated or control samples. The integrative genomic viewer (IGV) was used to visualize read alignment to portions of the genome.^95^

#### Identification of tardigrade mtSSBs

The identity of Rv07050 and BV898_11351 as mtSSBs was determined by reciprocal BLAST. The *H. exemplaris* genome (v3.1.5) has another annotated transcript, BV898_04979, with 94.6% sequence identity to the *H. exemplaris* mtSSB, BV898_11351 (Figures S1E and S1H).^27^ However, BV898_04979 had very limited coverage of library reads mapping to it, and we were unable to amplify it from either cDNA or genomic template (Figures S1F–S1I). Furthermore, expression of the hypothetical BV898_04979 protein did not confer the same desiccation survival benefits as the tardigrade mtSSBs (Figures S1J and S1K), suggesting that the annotated sequence differences of BV898_04979 reduce its function, at least in this heterologous context. Thus, we believe that *H. exemplaris*, like most other species, has a single mtSSB. Because BV898_04979 and BV898_11351 are near the ends of genomic scaffolds, it is possible that these scaffolds represent a contiguous genomic region with a single mtSSB.

#### Molecular cloning

To clone mtSSBs and other control proteins, sequences were codon optimized for *E. coli* (Integrated DNA Technologies). To clone non codon-optimized versions of *R. varieornatus* mtSSB and *H. exemplaris* mtSSB, RNA was isolated from tardigrades, converted to cDNA using the SuperScript III First-Strand Synthesis System (Invitrogen, Cat #18080-051) with oligo(dT) priming, and PCR amplified from total cDNA with gene-specific primers including 30bp of homology to pDEST17. pDEST17 was linearized by high fidelity PCR (Q5 High-Fidelity 2x Master Mix, NEB, Cat #M0492), during which sequence encoding the 6x His tag was removed. Sequences of primers used to linearize pDEST17 were TGATTCGAGGCTGCTAACAAAG and GTAGTACGACATATGTATATCTC. PCR products were gel-extracted and cleaned (Zymoclean Gel DNA Recovery Kit, Cat #D4002), and assembled into the pDEST17 backbone with NEBuilder HiFi Assembly Master Mix (NEB, Cat #E2621). Correct assembly of each plasmid was verified by sequencing (Genewiz/Azenta).

Other versions of proteins like mtSSB domain constructs and species’ ΔMTS versions were similarly assembled. To clone plasmids for expression of the OB fold domain of mtSSBs from various species, the NCBI conserved domain search tool was used to identify the OB fold of each protein.^96^ The region of each protein with homology to cd04496 (SSB_OBF) was cloned into pDEST17 for bacterial expression. To create expression constructs with individual amino acid substitutions, plasmids were edited via site-directed mutagenesis (Q5 Site-Directed Mutagenesis Kit, NEB, Cat #E0554).

#### Bacterial expression of proteins

Heterologous expression of proteins was carried out in *E. coli* BL21-AI (Invitrogen, Cat #C607003). Single colonies were grown overnight at 37°C in LB with ampicillin selection. Overnight cultures were diluted 1:20 into LB with ampicillin and 0.2% L-arabinose (Thermo scientific, Cat #A11921.18) to induce protein expression. Expression of GFP and mCherry was visually apparent, providing a simple visual confirmation of protein induction. To assess protein expression levels, protein was visualized with SDS-PAGE and Coomassie staining. Bacteria that were induced for 4 h at 37°C with 0.2% L-arabinose were pelleted in 1.5 mL tubes by centrifugation at 5,000 rpm for 10 min and resuspended in 200 μL of 0.85% NaCl. Bacteria were lysed with 30 pulses of sonication using a Branson Sonifier 250 set to 50% output and 50% duty cycle. To isolate the soluble fraction, total lysate was centrifuged at 14,000 rpm for 10 min at 4°C. Protein concentration in each fraction was determined with a Bio-Rad Protein Assay Kit (#5000002). 2 μg protein was combined with sample loading buffer (2x buffer = 4% SDS, 20% glycerol, 0.125 M Tris-HCl pH 6.8, 10% 2-mercaptoethanol, 0.004% bromphenol blue), heated at 95°C for 10 min, and loaded into wells of a NuPAGE 4–12% Bis-Tris mini protein gel (NP0322BOX). Samples were run alongside protein marker (Precision Plus Protein Kaleidoscope, Bio-Rad #1610375). Samples were run in MOPS SDS Running Buffer (Invitrogen, NP0001) for 70 min at 140 V. Gels were stained in Coomassie (0.1% Coomassie brilliant blue, 50% methanol, 40% water, 10% acetic acid) overnight at 4°C with rocking and destained in a 5:4:1 solution of water:methanol:acetic acid. Gels were imaged with a Bio-Rad Universal Hood II Molecular Imager using Image Lab 5.2.1 software. The expected molecular weights of proteins that are reported were computed with the Expasy Compute pI/Mw tool.^97^ Quantification of relative protein expression or solubility (Figure S2E) was carried out in FIJI by subtracting the background of gel images and taking the ratio of the integrated density of the band for each protein divided by the total integrated density of the lane.

#### Analysis of bacterial growth rate and length

We determined bacterial growth rates by measuring OD600 over time in a plate reader. Overnight cultures of bacteria were diluted 1:20 for induction in LB with ampicillin and 0.2% L-arabinose in a 96-well plate. Plates were kept at 37°C in a BioTek Synergy H1 plate reader with double orbital shaking. OD600 measurements were taken every 10 min. Three independent biological replicates were conducted. Length of bacteria was determined by imaging cells on a Nikon Eclipse E800 microscope with a pco.panda sCMOS camera and measuring lengths in FIJI.

#### Bacterial mRNA-seq

To test for transcriptional changes in *E. coli* expressing *R. varieornatus* mtSSB we conducted mRNA-seq. Briefly, total RNA was isolated from *E. coli* BL21-AI (Invitrogen, Cat #C607003) expressing either GFP or *R. va* mtSSB using a TRIzol Max Bacterial RNA Kit (Invitrogen, Cat #16096020). Bacteria were induced for 4 h, as described, and total RNA was isolated according to the kit instructions. Total RNA was sent to Novogene where ribosomal RNA was depleted (Ribo-ZeroTM) and libraries were prepared (NEB directional library kit) for 150 bp paired end sequencing on a Novaseq (Illumina). Reads were mapped to the genome of *E. coli* BL21-AI using bowtie2 and assigned to gene products with featurecounts. Differential expression of transcripts was calculated with EdgeR.^98,99^ Individual transcripts known to be associated with the SOS response were identified within this dataset.^40,43^

#### Bacterial desiccation, heat, and radiation survival

The OD600 of bacteria was measured after 4 h of induction with L-arabinose. The volume of bacteria to be desiccated was normalized to a concentration of 1.5 OD600 for each condition. Bacteria were collected by centrifugation (5,000 rpm, 10 min), washed with 1 mL 0.85% NaCl, and resuspended in 1 mL 0.85% NaCl. 0.85% NaCl is isotonic to the bacteria, so as not to cause osmotic stress to cells. A dilution series was plated onto LB + AMP plates to determine the concentration of bacteria before exposure to any stress condition. Bacteria were pelleted by centrifugation (5,000 rpm, 10 min) and the supernatant was removed before overnight (~16hr) desiccation at room temperature (~22°C) in a Savant speedvac concentrator. Bacteria were rehydrated in the same volume, and a dilution series was plated to determine the concentration of survivors. Survival was calculated as the colony forming units (cfu) after desiccation divided by that of controls. Bacterial desiccation survival was also measured following drying in a desiccation chamber (Figure S8). In this experiment, bacteria were prepared as with experiments with drying in the speedvac, but were instead transferred to a desiccation chamber with Drierite desiccant (Fisher, Cat #07-578-4A). In each case, the bacteria were exposed to a relative humidity of ~3–4%. To assess survival of heat shock and radiation, the same protocol was followed except with exposure to 52°C for 1 h or to 2,180 Gy irradiation. These stress conditions were chosen based on previous optimization.^30,56^

#### Yeast expression and imaging

Expression constructs carrying mtSSBs, or parts thereof, were codon optimized for yeast. These fragments were synthesized (Integrated DNA Technologies) with overhangs for BP reactions (Invitrogen BP clonase II, Cat #11789-020) to insert constructs into pDONR221. Subsequent LR reactions (Invitrogen LR clonase II, Cat #11791-020) moved constructs from pDONR221 into pAG415. Cloning reactions were carried out per kit instructions. Plasmids were sequence-verified to ensure correct assemblies.

Plasmids were transformed into *S. cerevisiae* (W303) MATa ura3-1, leu2-3,112, his3-11, trp1-1, can1-100, ade2-1. Briefly, a 5 mL overnight culture of yeast was grown in yeast bacto-peptone dextrose (YPD) at 24°C in an orbital shaker. 0.5 mL of overnight culture was added to 25 or 50 mL of culture and grown to log phase (OD660 0.3–0.8) with shaking. Yeast cells were pelleted by centrifugation and resuspended in 5 mL of TE Lithium acetate. Cells were again concentrated by centrifugation and most of the supernatant was removed. Yeast in minimal remaining supernatant were kept on ice for 15 min. Salmon sperm DNA (Agilent technologies, Cat #201190) was boiled for 5 min and then stored on ice. For each transformation, 50 μL of cells, 5 μL of salmon sperm DNA, and 1 μL of expression plasmid were combined in an Eppendorf tube. To each tube, 250 μL of TE/lithium acetate/PEG was added. Samples were incubated at 32°C with shaking for 30 min and heated to 42°C for 15 min. Cells were then pelleted by centrifugation, resuspended in 250 μL 1M sorbitol, and plated onto SD -LEU plates for selection.

The endogenous Cox4mCherry reporter strain was created by fusing endogenous Cox4 sequence to an mCherrykanMX selectable marker (pFA6) by PCR. The following primers were used to amplify this sequence: GAATGTGGTTCTGTTTACAAACTAAACCCTGTTGGTGTTCCAAATGATGACCACCATCACcggatccccgggttaattaa, AATAAAGAAGAAGGTAAAAAGTAAAAGAGAAACAGAAGGGCAACTTGAATGATAAGATTAGaattcgagctcgtttaaac. The PCR product was purified (Zymoclean Gel DNA Recovery Kit, Cat #D4002) and transformed into *S. cerevisiae* W303 following the same protocol as with plasmids, except that cells were kept on a roller drum at 24°C for 18 h outgrowth in YPD before plating on YPD with 2x G148 for selection.

To visualize protein localization, yeast were grown overnight in SG -LEU with adenine (0.5 mg/mL) for induction (24°C with shaking). Yeast not carrying the Cox4mCherry reporter were stained with Mitotracker Red FM (Invitrogen, M22425) to confirm localization patterns. Cells induced overnight in SG -LEU with adenine were isolated and stained in 50 μM Mitotracker Red and 0.1 M HEPES in yeast extract plus casamino acids (YC complete, no sugar) for 30 min. Cells were washed and resuspended in YC complete without sugar for imaging. Cells with GFP- and mCherry-labeled proteins or Mitotracker staining were imaged with a Nikon TiE base with CSU-X1 spinning disk head (Yokogawa), and ImagEM EMCCD camera (Hamamatsu) using Metamorph software for image acquisition.

To stain yeast with DAPI, cells were first washed in YC complete without sugar. Cells were then fixed in 4% paraformaldehyde (Electron Microscopy Sciences, Cat #15710) in PBS for 1 h with rocking. Cells were mounted on glass slides in a 1:1 ratio of YC complete:DAPI Fluoromount-G (SouthernBiotech, Cat #0100-20). Yeast were imaged with a Zeiss 880 LSM with fast Airyscan.

#### Yeast desiccation survival

To measure desiccation survival, tardigrade mtSSBs without fluorescent labels and control proteins were transformed into yeast. Protein expression was induced and mid-log phase (OD660 0.4–0.6) cells were concentrated for desiccation. Dilution series’ of controls and samples after overnight (~16hr) desiccation in a speedvac at room temperature and 1 h rehydration in SD -LEU liquid media were plated (onto SD -LEU) to determine cfu concentrations. Survival was calculated as the ratio of cfu after desiccation divided by that of controls.

#### Sequence alignment and MTS prediction

Predictions of mitochondrial targeting sequences from mtSSBs were generated with TargetP2.0.^100^ Protein sequences for mtSSBs and *E. coli* SSB were aligned with MUSCLE. The predicted mitochondrial targeting sequences were removed for alignments. The alignment was visualized with Jalview and colored according to sequence identity.

#### Protein structure prediction

The structure of *R. varieornatus* mtSSB ΔMTS was predicted using alphafold colab v2.3.2.^59^ The multimer model was used with an input of four copies of the *R. varieornatus* mtSSB ΔMTS. We also confirmed that introduction of DNA-binding mutations to W91 and F97 did not significantly disrupt predicted protein structure.

#### Protein purification

Sequence encoding 6X HisTEV was added to the bacterial expression construct carrying *R. va* mtSSB ΔMTS by PCR amplification and ligation (Q5 Site-Directed Mutagenesis Kit, NEB, Cat #E0554). The plasmid insert was sequence-verified and then transformed into *E. coli* BL21-AI (Invitrogen, C607003). Single colonies were picked into 50 mL LB with ampicillin for overnight culture at 37°C with shaking. Overnight cultures were diluted 1:20 into a total volume of 2 L of LB with ampicillin and 0.2% L-arabinose. Protein expression was induced for 6 h, at which point bacteria were harvested by centrifugation (5,000 rpm, 10 min, at 4°C). Cell pellets were washed in 0.85% NaCl and frozen at −80°C.

The cell pellet was thawed in Talon buffer A (50 mM sodium phosphate pH 7.4, 300 mM NaCl, 5 mM imidazole) with 50 μg/mL lysozyme, 12.5 U/mL DNase, and 1x protease inhibitors (Roche). Resuspended cells were sonicated then centrifuged at 12,000 xg for 30 min. Soluble lysate was 0.45 μm filtered then loaded onto 1 mL Talon Crude columns (Cytiva). After washing to baseline with buffer A, bound protein was eluted with Talon buffer B (50 mM sodium phosphate pH 7.4, 300 mM NaCl, 250 mM imidazole).

Eluate from Talon affinity chromatography was collected and dialyzed against 20 mM Tris HCl pH 7.4, 150 mM NaCl. TEV protease was added at a 10:1 target:protease mass ratio while in dialysis and left to incubate at 4°C overnight. The digest at this stage had a volume of approximately 3 mL and was diluted to approximately 25 mL in Talon buffer A to facilitate column loading. The samples were loaded on 1 mL Talon Crude columns with the flowthroughs collected as ‘released protein.’ Flowthroughs were concentrated to approximately 500 μL, and all samples were analyzed with SDS PAGE.

#### Gel shift assays

Gel shift assays were carried out as previously described.^62,63^ Purified *R. varieornatus* ΔMTS mtSSB was combined with 4.7 nM M13mp18 ssDNA (NEB, N4040S) at ratios calculated with the following equation: R = n × ([mtSSB tetramer]/[nucleotide]), using *n* = 33 nucleotides/tetramer. Protein and DNA were incubated together for 1 h at 22°C in 30 μL of buffer T (10 mM Tris (pH8.1), 0.1 mM EDTA) with additions of either 20 mM NaCl or 300 mM NaCl. 2 μL of loading dye (50% glycerol, 0.04% bromophenol blue) was added to each reaction and loaded into wells of a 0.5% agarose gel. Gels were run in 20 mM Tris, 0.4 mM sodium acetate, 0.2 mM EDTA running buffer for 3 h at 60 V, then soaked in buffer T with 1 M NaCl for 1 h at 4°C. Gels were stained for 30–60 min in 2 μg/mL ethidium bromide and destained for 1 h in buffer T with 1 M NaCl, also at 4°C. Gels were imaged with a Bio-Rad Universal Hood II Molecular Imager and Image Lab 5.2.1 software. Images were inverted for display in figures (Figures 2B and 2C).

#### Phylogenetic tree construction

MEGAX was used to create a phylogenetic tree of replication protein A (RPA) and mtSSB homologs.^101,102^ The following protein sequences were obtained from NCBI: NP_002936.1, NP_001284487.1, NP_002938.1, XP_005250105.1, AAH19119.1, AAH04578.1, AAH28489.1, NP_001273592.1, NP_524274.1, NP_001260652.1, NP_001285131.1, NP_536744.2, KZV13472.1, CAA96241.1, CAA89468.1, NP_009958.2, WP_089642251.1. The following protein sequences were obtained from Wormbase: *C. elegans* RPA-1, RPA-2, RPA-4a, and MTSS-1. The following tardigrade homologs were identified via BLAST and protein sequences obtained from *H. exemplaris* genome annotation v3.1.5, and *R. varieornatus* genome Rv1: BV898_09830, BV898_02372, BV898_10131, BV898_11351, RvY_18611-1, RvY_00174-1, RvY_10449-1, RvY_07050-1.^27^ Full length sequences (including mitochondrial targeting sequences of mtSSBs) were aligned with MUSCLE. The evolutionary history was inferred by using the Maximum Likelihood method and JTT matrix-based model.^103^ The percentage of replicate trees in which the associated taxa clustered together in the bootstrap test (500 replicates) are shown next to the branches.^104^ Initial tree(s) for the heuristic search were obtained automatically by applying Neighbor-Join and BioNJ algorithms to a matrix of pairwise distances estimated using the JTT model, and then selecting the topology with superior log likelihood value. There were 859 positions in the final dataset.

### QUANTIFICATION AND STATISTICAL ANALYSIS

Enrichment of individual tardigrade genes following selection of bacterial expression cloning screens was determined by analysis with DESeq2 in order to account for variability and assign FDR values to assess significance.^93^ For survival experiments in bacteria and yeast, we first performed a 1-way ANOVA to test for differences across all conditions. If this produced a significant result, we then conducted post-hoc Tukey tests to define pairwise differences. The number of independent biological replicates is indicated in figure legends or in the text. Statistical differences in bacterial length were assessed with Dunnett’s test, using GFP-expressing bacteria as the control group. In each case 100–200 individual bacterial cells were sampled. Statistical analyses were carried out in Rstudio.

## Supplementary Material

1

2

## Figures and Tables

**Table:** KEY RESOURCES TABLE

REAGENT or RESOURCE	SOURCE	IDENTIFIER
Bacterial and virus strains		
ElectroMAX DH10B T1 Phage Resistant Cells	Invitrogen	12033015
BL21-AI *E. coli*	Invitrogen	C607003
Chemicals, peptides, and recombinant proteins		
Agar	Fisher Scientific	BD214050
Kanamycin	Sigma	K-1377
Ampicillin	Sigma	A0166
Gibco bacto tryptone	Fisher Scientific	DF0123-17-3
Sodium Chloride	Fisher Scientific	S641-212
Gibco yeast extract	Fisher Scientific	DF0127-17-9
L-arabinose	Thermo Fisher	A11921.18
Paraformaldehyde	Electron Microscopy Sciences	15710
Coomassie brilliant blue	Gibco	15528–011
Methanol	Sigma	179337-2L
Acetic acid	Fisher Scientific	A38-212
Critical commercial assays		
PicoPure RNA isolation kit	Applied Biosystems	KIT0204
Cloneminer II cDNA Library Construction Kit	Invitrogen	A11180
HiPure Plasmid Filter Midiprep Kit	Invitrogen	K210014
Purelink HQ Plasmid Purification Kit	Invitrogen	K2100-01
Kapa2G Robust Hotstart PCR Kit	Roche	07961073001
ChargeSwitch-Pro PCR cleanup kit	Invitrogen	CS32050
SuperScript III First-Strand Synthesis System	Invitrogen	18080–051
Q5 High-Fidelity 2x Master Mix	NEB	M0492
Gel DNA Recovery Kit	Zymo	D4002
NEBuilder HiFi Assembly Master Mix	NEB	E2621
Q5 Site-Directed Mutagenesis Kit	NEB	E0554
NuPAGE^™^ Bis-Tris Mini Protein Gels, 4–12%, 1.0–1.5 mm	Thermo Fisher	NP0322BOX
Protein Assay Kit	Bio-Rad	5000002
TRIzol Max Bacterial RNA Kit	Invitrogen	16096020
BP clonase II	Invitrogen	11789–020
LR clonase II	Invitrogen	11791–020
Deposited data		
Expression cloning sequencing data	This study	PRJNA1008366
Bacterial RNA-seq data	This study	PRJNA1070671
Experimental models: Organisms/strains		
*Hypsibius exemplaris* (Z151)	Carolina Biological	133960
*Ramazzottius c.f. varieornatus* (YOKOZUNA-1)	Gift from K. Arakawa	N/A
*Chlorococcum* sp.	Carolina Biological	152091
Super Fresh Chlorella V12	Reed Mariculture	N/A
S. *cerevisiae* (W303) MATa ura3-1, leu2-3,112, his3-11, trp1-1, can1-100, ade2-1	American Tissue Culture Connection	208352
Oligonucleotides		
cDNA insert amplification forward: CATCACCATCACCTCGAATCAAC	Integrated DNA Technologies	N/A
cDNA insert amplification reverse: TTCGGGCTTTGTTAGCAGCCTCGAATC	Integrated DNA Technologies	N/A
pDEST17 linearize forward: TGATTCGAGGCTGCTAACAAAG	Integrated DNA Technologies	N/A
pDEST17 linearize reverse: GTAGTACGACATATGTATATCTC	Integrated DNA Technologies	N/A
Recombinant DNA		
pDONR222	Invitrogen	Included with A11180
pDEST17	Invitrogen	11803012
pDONR221	Invitrogen	12536017
pAG415	Addgene	Plasmid #14146
pFA6	Addgene	Plasmid #39296
M13mp18 ssDNA	NEB	N4040S
Software and algorithms		
Bowtie2	https://bowtie-bio.sourceforge.net/bowtie2/index.shtml	N/A
Featurecounts	https://subread.sourceforge.net/featureCounts.html	N/A
DEseq2	https://bioconductor.org/packages/release/bioc/html/DESeq2.html	N/A
Integrative genomic viewer	https://igv.org/	N/A
Image Lab 5.2.1	Bio-Rad	N/A
FIJI	https://imagej.net/software/fiji/	N/A
EdgeR	https://bioconductor.org/packages/release/bioc/html/edgeR.html	N/A
RStudio	https://cran.r-project.org/https://posit.co/download/rstudio-desktop/#download	N/A
Jalview	https://www.jalview.org/download/	N/A
MEGAX	https://www.megasoftware.net/	N/A
Other		
Vented 35 mm petri dishes	Tritech Research	T3500
Deer Park spring water	Staples	705032
Aspirator tube assembly	Millipore Sigma	A5177
Borosilicate glass capillary	World Precision Instruments	1B100F-4
Electroporator	Eppendorf	2510
S.O.C. media	Invitrogen	15544034
Speedvac	Savant	
Drierite desiccant	Fisher Scientific	07-578-4A
Branson Sonifier 250	Branson	SFX250
Precision Plus Protein Kaleidoscope	Bio-Rad	1610375
MOPS SDS Running Buffer	Invitrogen	NP0001
Biotek Synergy H1 multimode reader	Biotek	
Salmon sperm DNA	Agilent technologies	201190
Mitotracker Red FM	Invitrogen	M22425
DAPI Fluoromount-G	Southern Biotech	0100–20

## Highlights

- Bacterial expression cloning is an effective method to identify desicco-protectant proteins
- Mitochondrial single-stranded DNA-binding proteins are potent desicco-protectants in bacteria
- mtSSBs promote desiccation tolerance by binding to DNA
