# Supplementary material for: A bacterial expression cloning screen reveals single-stranded DNA-binding proteins as potent desicco-protectants
Source: Cell Rep. Author manuscript; Available in PMC 2024 Dec 18. (PMC11654893; doi:10.1016/j.celrep.2024.114956)
Supplement: 1 [file NIHMS2038877-supplement-1.pdf]

Cell Reports, Volume *43*

Supplemental information

A bacterial expression cloning screen reveals single-stranded DNA-binding proteins as potent desicco-protectants

Jonathan D. Hibshman, Courtney M. Clark-Hachtel, Kerry S. Bloom, and Bob Goldstein

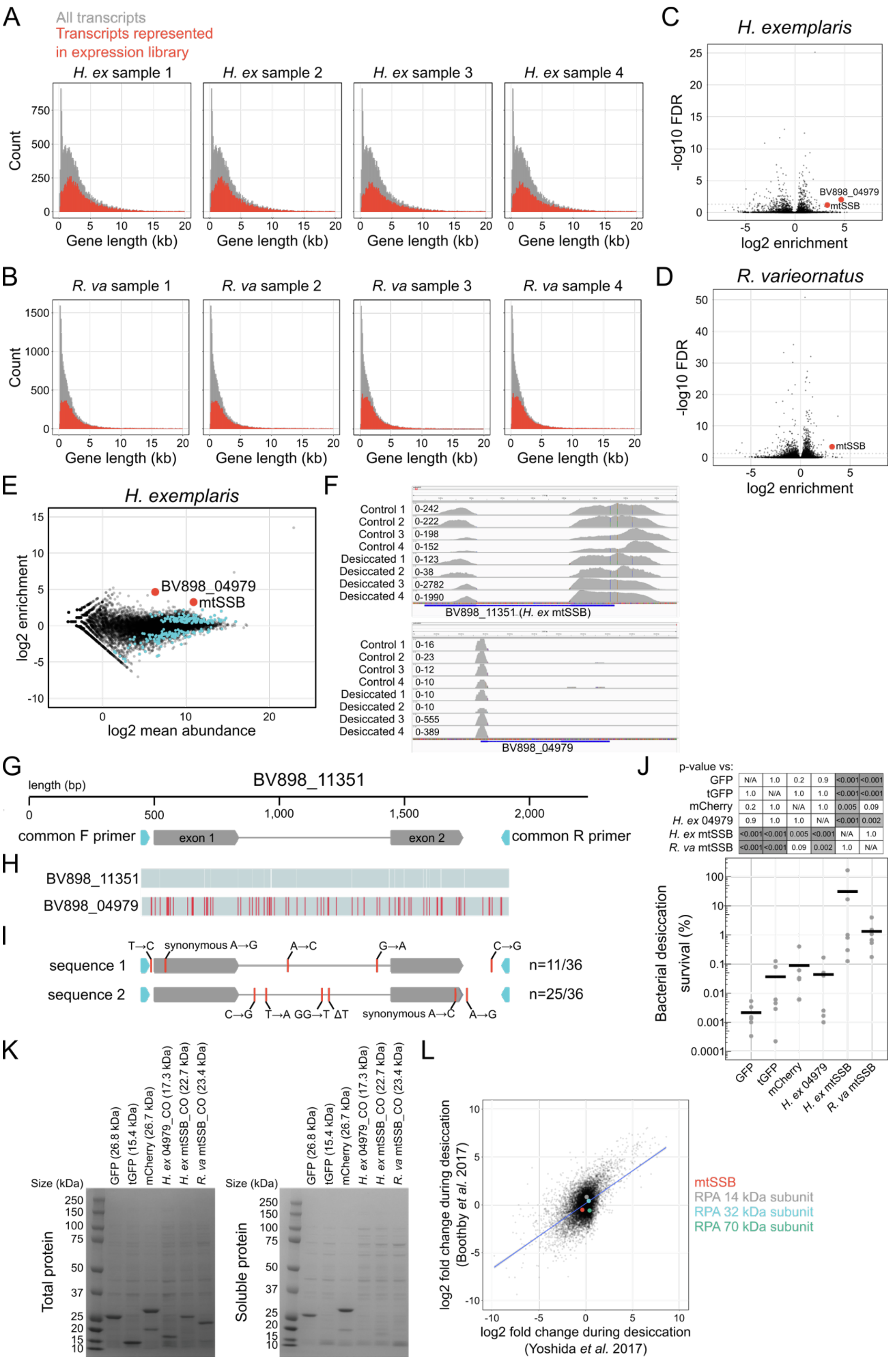

p-value vs:						
GFP	N/A	1.0	0.2	0.9	<0.001	<0.001
tGFP	1.0	N/A	1.0	1.0	<0.001	<0.001
mCherry	0.2	1.0	N/A	1.0	0.005	0.09
H. ex 04979	0.9	1.0	1.0	N/A	<0.001	0.002
H. ex mtSSB	<0.001	<0.001	0.005	<0.001	N/A	1.0
R. va mtSSB	<0.001	<0.001	0.09	0.002	1.0	N/A

Figure S1. Controls for expression cloning screens and evidence for a single mtSSB in *Hypsibius exemplaris*. A,B) Representation of transcripts in each of four replicates of the expression libraries derived from *H. exemplaris* (A) or *R. varieornatus* (B). Any transcript with a read mapped to it is indicated in red versus the background (gray) showing all transcripts in the genome. **C,D)** Volcano plots of -log10 FDR vs log2 fold enrichment of cDNAs from expression cloning screens in *H. exemplaris* (C) and *R. varieornatus* (D). **E)** The same plot as in Figure 1B, also highlighting BV898_04979 in addition to BV898_11351 (mtSSB). **F)** Distribution of reads aligned to BV898_04979 and BV898_11351. Integrative genomics viewer was used to visualize aligned reads. **G)** Annotation of the genomic locus of BV898_11351. This sequence is found on scaffold0105. BV898_11351 contains two exons. Common primers were designed in a region of identical sequences between the flanking genomic regions of BV898_04979 (scaffold0025) and BV898_11351 (scaffold0105). **H)** An alignment of genomic loci of BV898_11351 (scaffold0105) and BV898_04979 (scaffold0025) reveals significant sequence differences between the two scaffolds. **I)** Amplification and sequencing of genomic DNA from individual tardigrades revealed the presence of two versions of BV898_11351. Each of these had some polymorphisms relative to the consensus scaffold sequence. In no case was genomic sequence from scaffold0025 (BV898_04979) identified. The frequency of each sequence was derived from sequencing six clones per individual for six tardigrades. **J)** Expression of BV898_04979 did not significantly improve bacterial desiccation survival. Note, data for GFP, tGFP, mCherry, *H. ex* mtSSB, and *R. va* mtSSB are the same as in Figure 1F as *H. ex* BV898_04979 was tested in parallel to these controls. The p-values reported are calculated by Tukey's test on the entire panel of strains, inclusive of *H. ex* BV898_04979. Data points represent individual replicates and bars indicate mean survival values. **K)** SDS-PAGE analysis of bacterial protein expression (total and soluble) reveals strong expression and solubility of fluorophore controls. BV898_04979 and mtSSBs were well-expressed, but had limited solubility. **L)** Expression changes of *H. exemplaris* transcripts during desiccation are plotted on a graph comparing log2 fold change values from Boothby *et al.* 2017 to those of Yoshida *et al.* 2017. *H. exemplaris* mtSSB and replication protein A homologs do not display significant transcriptional changes during desiccation in tardigrades.

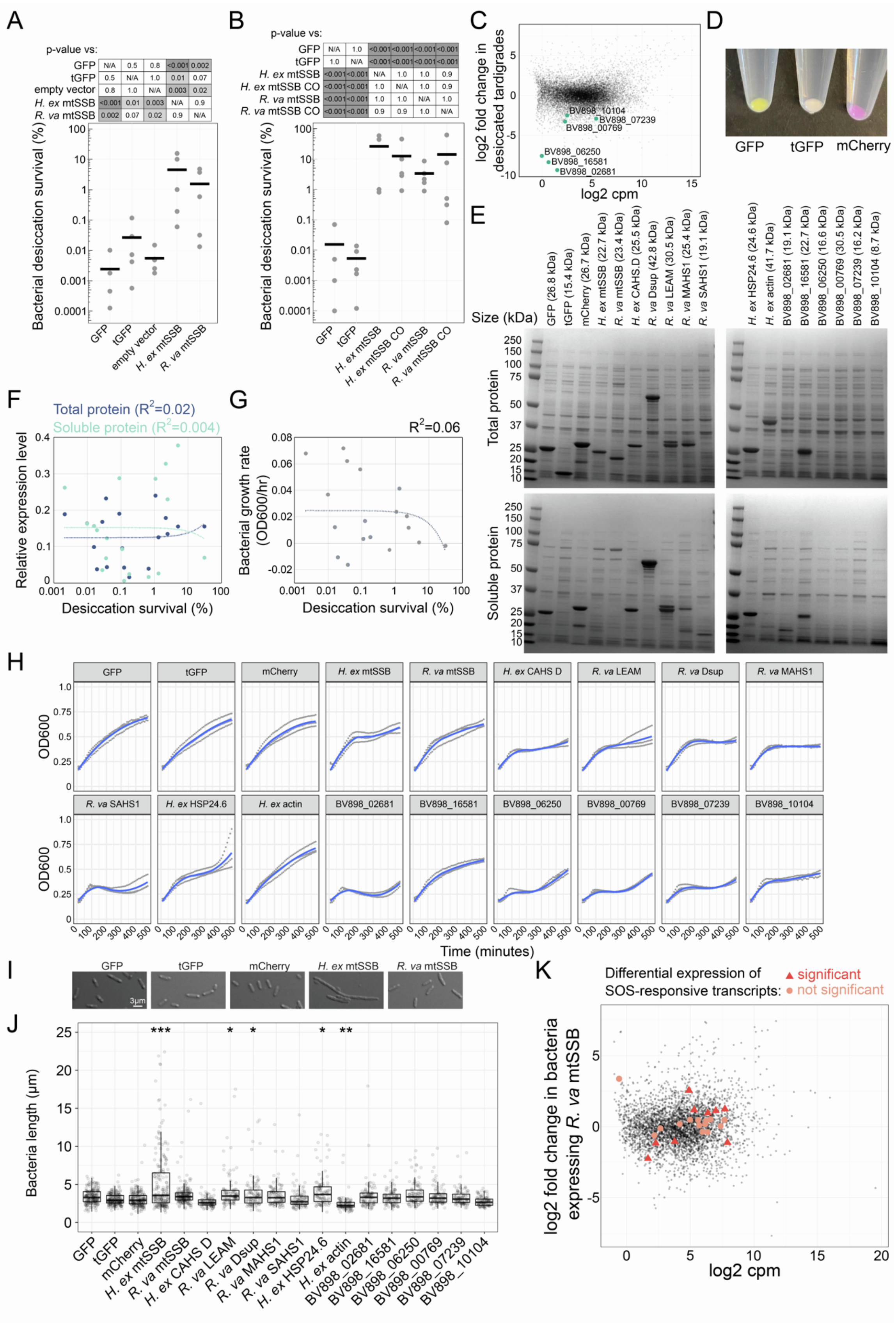
A
p-value vs:
GFP N/A 0.5 0.8 <0.001 0.002
tGFP 0.5 N/A 1.0 0.01 0.07
empty vector 0.8 1.0 N/A 0.003 0.02
H. ex mtSSB <0.001 0.01 0.003 N/A 0.9
R. va mtSSB 0.002 0.07 0.02 0.9 N/A
Bacterial desiccation survival (%)
GFP
tGFP
empty vector
H. ex mtSSB
R. va mtSSB
B
p-value vs:
GFP N/A 1.0 <0.001 <0.001 <0.001 <0.001
tGFP 1.0 N/A <0.001 <0.001 <0.001 <0.001
H. ex mtSSB <0.001 <0.001 N/A 1.0 1.0 0.9
H. ex mtSSB CO <0.001 <0.001 1.0 N/A 1.0 0.9
R. va mtSSB <0.001 <0.001 1.0 1.0 N/A 1.0
R. va mtSSB CO <0.001 <0.001 0.9 0.9 1.0 N/A
Bacterial desiccation survival (%)
C
log2 fold change in desiccated tardigrades
log2 cpm
BV898_10104
BV898_07239
BV898_00769
BV898_06250
BV898_16581
BV898_02681
D
GFP
tGFP
mCherry
E
Size (kDa)
GFP (26.8 kDa)
tGFP (15.4 kDa)
mCherry (26.7 kDa)
H. ex mtSSB (22.7 kDa)
R. va mtSSB (23.4 kDa)
H. ex CAHS.D (25.5 kDa)
R. va Dsup (42.8 kDa)
R. va LEAM (30.5 kDa)
R. va MAHS1 (25.4 kDa)
R. va SAHS1 (19.1 kDa)
H. ex HSP24.6 (24.6 kDa)
H. ex actin (41.7 kDa)
BV898_02681 (19.1 kDa)
BV898_16581 (22.7 kDa)
BV898_06250 (16.6 kDa)
BV898_00769 (30.5 kDa)
BV898_07239 (16.2 kDa)
BV898_10104 (8.7 kDa)
Total protein
Soluble protein
F
Total protein (R²=0.02)
Soluble protein (R²=0.004)
Relative expression level
Desiccation survival (%)
G
R²=0.06
Bacterial growth rate (OD600/hr)
Desiccation survival (%)
H
GFP
tGFP
mCherry
H. ex mtSSB
R. va mtSSB
H. ex CAHS D
R. va LEAM
R. va Dsup
R. va MAHS1
R. va SAHS1
H. ex HSP24.6
H. ex actin
BV898_02681
BV898_16581
BV898_06250
BV898_00769
BV898_07239
BV898_10104
OD600
Time (minutes)
I
GFP
tGFP
mCherry
H. ex mtSSB
R. va mtSSB
3μm
J
Bacteria length (μm)
***
*
*
*
**
GFP
tGFP
mCherry
H. ex mtSSB
R. va mtSSB
H. ex CAHS D
R. va LEAM
R. va Dsup
R. va MAHS1
R. va SAHS1
H. ex HSP24.6
H. ex actin
BV898_02681
BV898_16581
BV898_06250
BV898_00769
BV898_07239
BV898_10104
K
Differential expression of SOS-responsive transcripts:
▲ significant
● not significant
log2 fold change in bacteria expressing R. va mtSSB
log2 cpm

Figure S2. Controls for validation of tardigrade mtSSBs as desiccation protectants. A) *E. coli* carrying an empty vector survive desiccation at a similar rate as GFP- and truncated GFP (tGFP)- expressing controls. Bacteria expressing tardigrade mtSSBs had significantly higher rates of desiccation survival relative to the empty vector control. **B)** Comparison of codon optimized (CO) and non-codon optimized versions of *H. exemplaris* mtSSB and *R. varieornatus* mtSSB reveal negligible differences in bacterial desiccation survival. P-values reported in A and B were derived from Tukey tests. Individual data points represent independent replicates and black lines depict mean values. **C)** A plot of transcriptional changes during desiccation in tardigrades from Boothby *et al.* 2017 was used to identify likely negative control genes that were less abundant during desiccation. **D)** Expression of fluorophores in bacteria provides a facile "tube-level phenotype" to confirm protein expression in each experiment. **E)** SDS-PAGE analysis of total and soluble protein from bacteria after 4 hrs of induction. **F)** Levels of total protein expression and soluble protein in bacteria were not correlated with desiccation survival. **G)** The slope from bacterial growth curves between 3.5 and 4.5 hrs (H) was not strongly correlated with desiccation survival. R^2 values from linear regressions are reported in E and F. Note, desiccation survival is plotted on a log axis in F and G. **H)** Growth curves for 8 hrs of culture are shown for bacteria with induced expression of each heterologous protein. Three replicates are shown in gray and a fitted curve is shown in blue. Cultures were harvested at 4 hr (240 min) for all desiccation and control experiments. **I)** Representative images of bacteria expressing GFP, tGFP, mCherry, *H. exemplaris* mtSSB, and *R. varieornatus* mtSSB show long filamentous bacteria resulting from expression of *H. exemplaris* mtSSB. **J)** The length of bacteria heterologously expressing mtSSBs and control proteins is plotted (n=100). Filamentous growth of bacteria is a hallmark of the SOS DNA damage response. Expression of *H. exemplaris* mtSSB caused a shift towards more filamentous growth, suggesting that the SOS response may be activated. The y-axis is limited to display most data points, but bacteria expressing *R. va* Dsup and *R. va* SAHS1 each had one outlier above the upper y-limit. Asterisks indicate a significant difference in length relative to GFP-expressing bacteria (Dunnett's test). * $p<0.05$, ** $p<0.01$, *** $p<0.001$. **K)** mRNA-seq of *E. coli* expressing GFP and *R. va* mtSSB reveals differential expression of many genes (gray), but limited upregulation of SOS response genes. Significantly changed SOS-responsive transcripts are indicated with red triangles and SOS-responsive transcripts that were not differentially expressed are shown as pink circles.

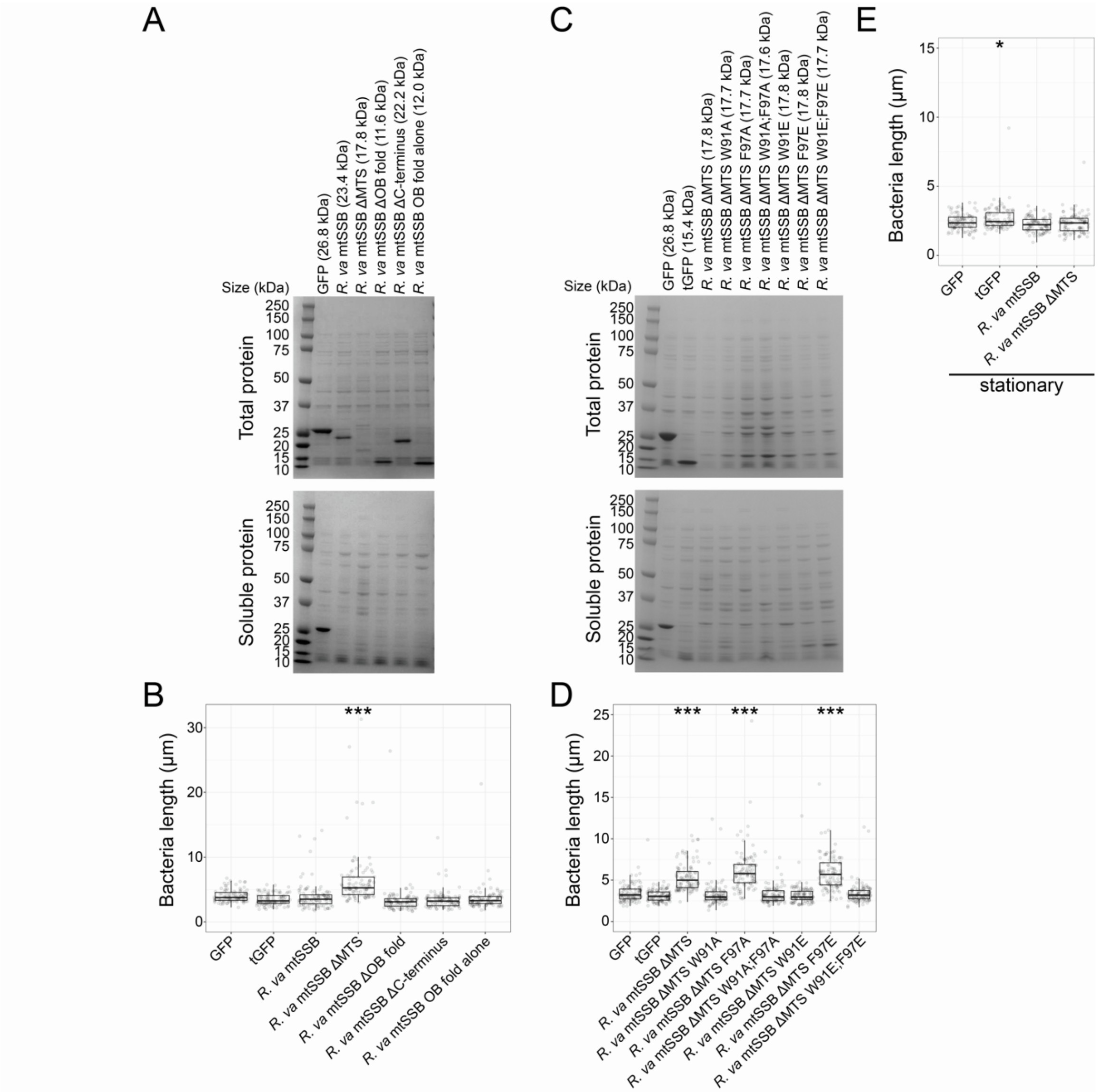

Figure S3. Analysis of expression and solubility of heterologous proteins and their impact on bacterial length. A) Total and soluble protein from bacteria expressing GFP or domains of *R. varieornatus* mtSSB. **B)** Length of bacteria expressing each of the constructs in A is plotted (n=100). **C)** Total and soluble protein from bacteria expressing *R. varieornatus* mtSSB with point mutations likely to disrupt DNA binding affinity. **D)** Length of bacteria expressing each of the constructs in C is shown (n=100). **E)** Length of bacteria in stationary phase expressing GFP, tGFP, *R. va* mtSSB, or *R. va* mtSSB ΔMTS is shown (n=100). P-values in B and D indicate a significant difference relative to bacteria expressing GFP. Similarly, in E, significant differences are reported relative to bacteria in stationary phase expressing GFP (Dunnett's test). * $p<0.05$, ** $p<0.01$, *** $p<0.001$.

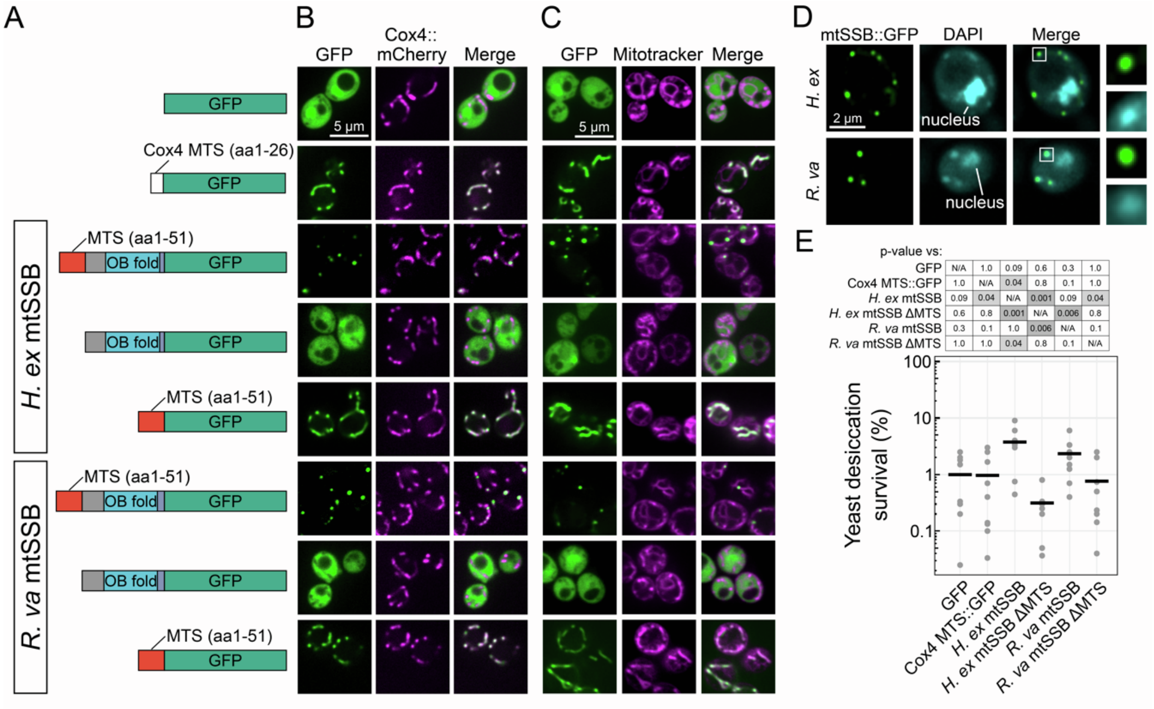

Figure S4. mtSSBs can enter mitochondria and offer modest desicco-protection to yeast. A) Schematic of yeast expression constructs used to assess protein localization. **B)** Co-expression of GFP-tagged proteins with endogenous Cox4::mCherry shows mitochondrial localization of tardigrade mtSSBs. **C)** Expression of GFP-tagged mtSSBs and co-staining with Mitotracker reveals punctate mitochondrial localization of *H. exemplaris* mtSSB and *R. varieornatus* mtSSB. **D)** *H. exemplaris* and *R. varieornatus* mtSSBs co-localize with DAPI-stained mtDNA. Boxes within merged images indicate regions shown to the right at 3.08x magnification. **E)** Mitochondrial localization is required for mtSSBs to improve yeast desiccation survival. P-values reported were calculated with a Tukey test (n=9).

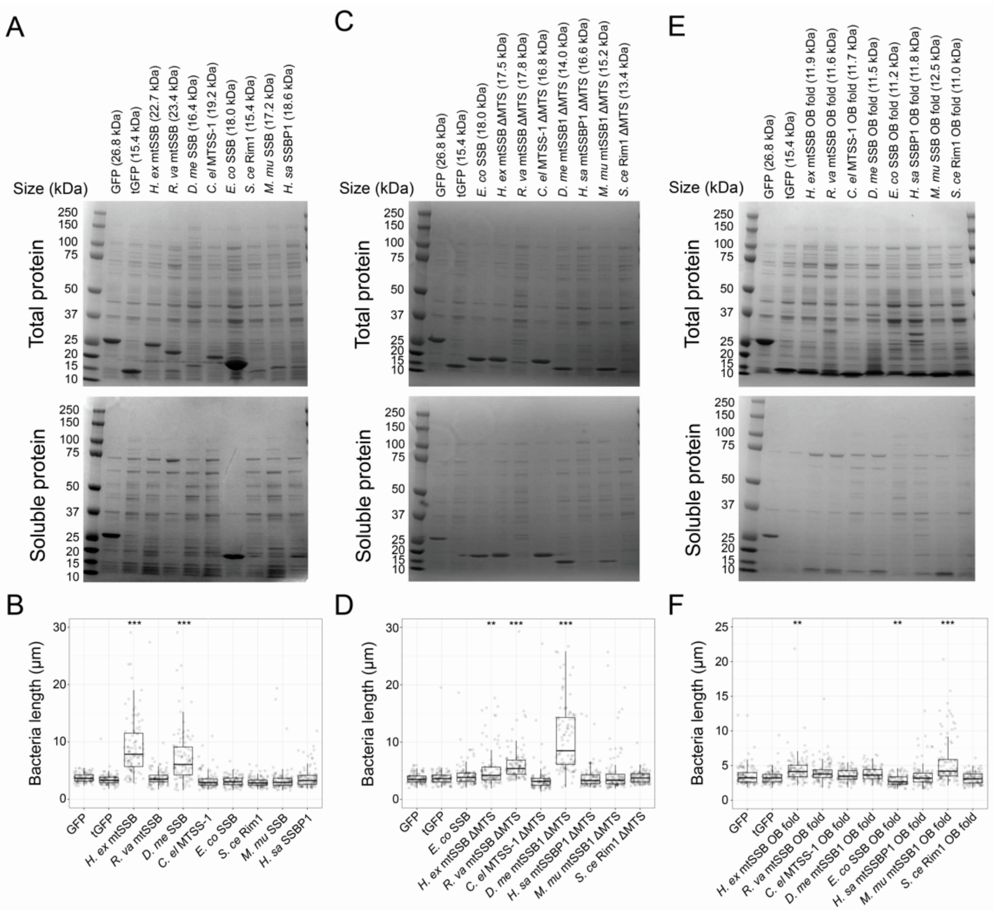

Figure S5. Analysis of bacterial expression levels and solubility of mtSSBs from various species and the effect of their expression on bacterial length. A) SDS-PAGE analysis reveals expression of each full-length protein in total bacterial lysate and some variability in solubility of these proteins. **B)** Length of bacteria expressing mtSSBs from various organisms is plotted. The y-axis was truncated to display data and excludes 3 outlier data points. **C)** SDS-PAGE analysis shows expression of mtSSBs lacking mitochondrial targeting sequences. The soluble fractions of the lysate were also run on a gel. **D)** Length of bacteria expressing mtSSBs without the mitochondrial target sequences is plotted. The y-axis was truncated to display data and excludes 4 outliers. **E)** Expression of OB fold domains alone was visualized from total and soluble fractions of bacterial lysate. **F)** Length of bacteria expressing the OB fold of various mtSSBs is shown. Statistics in B, D, and F were calculated with Dunnett's tests and asterisks indicate significance relative to GFP-expressing controls. ** p<0.01, *** p <0.001.

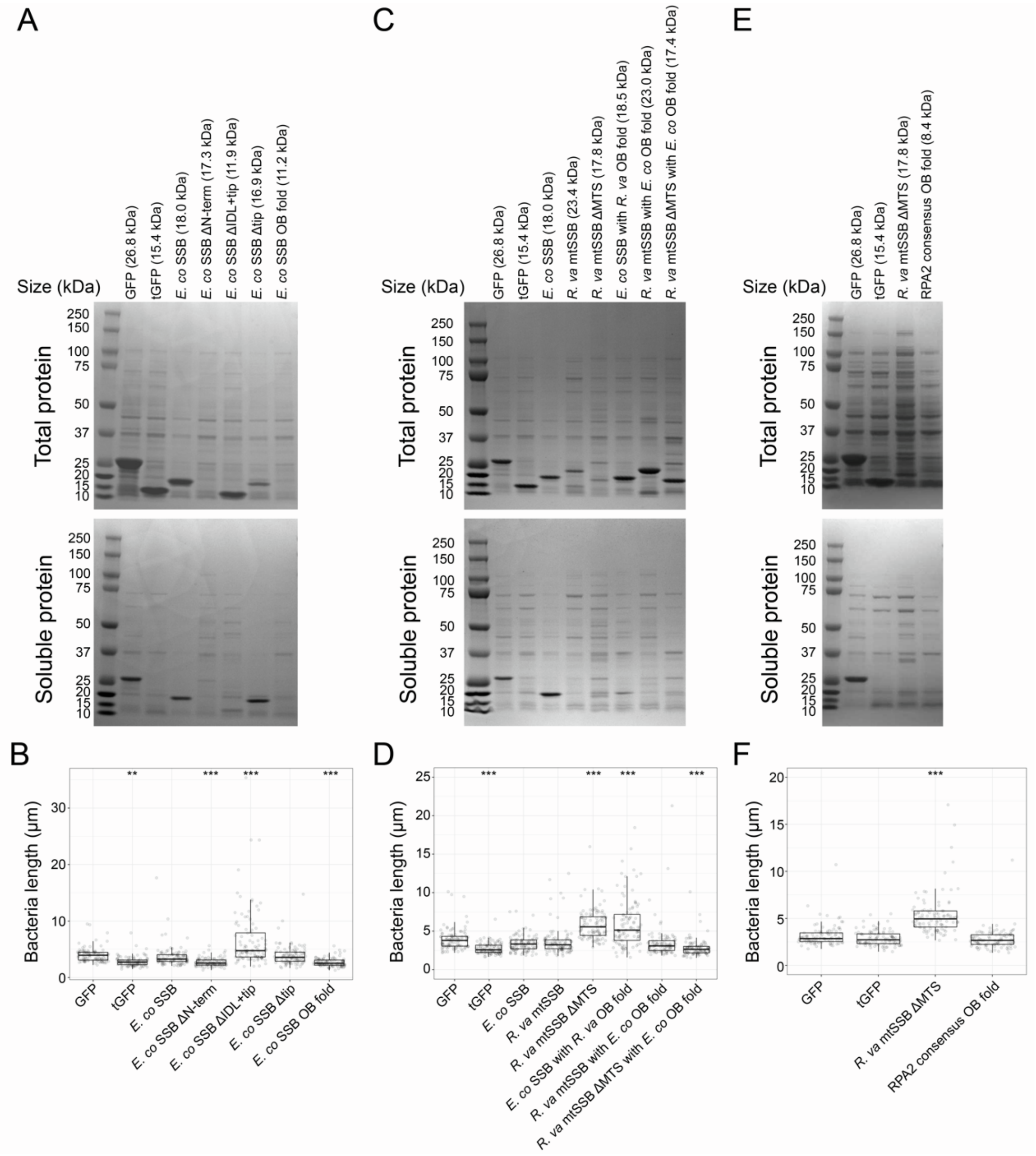

Figure S6. Controls associated with figure 4 analyzing expression and solubility of heterologous proteins and their effect on bacterial length. A) Control gels show expression levels and solubility of proteins comprised of different regions of the *E. co* SSB. **B)** Length of bacteria expressing each of the proteins in A. **C)** Control gels show levels of expression and solubility of chimeric *E. co* SSB/*R. va* mtSSB proteins. **D)** Length of bacteria expressing each of the proteins in C is plotted. **E)** Control gels for expression and solubility of the consensus RPA2 OB fold. **F)** Length of bacteria expressing control proteins and the RPA2 consensus OB fold is plotted. Significant differences in bacterial length relative to GFP-expressing controls in B, D, and F were determined with Dunnett's test. ** $p<0.01$, *** $p <0.001$.

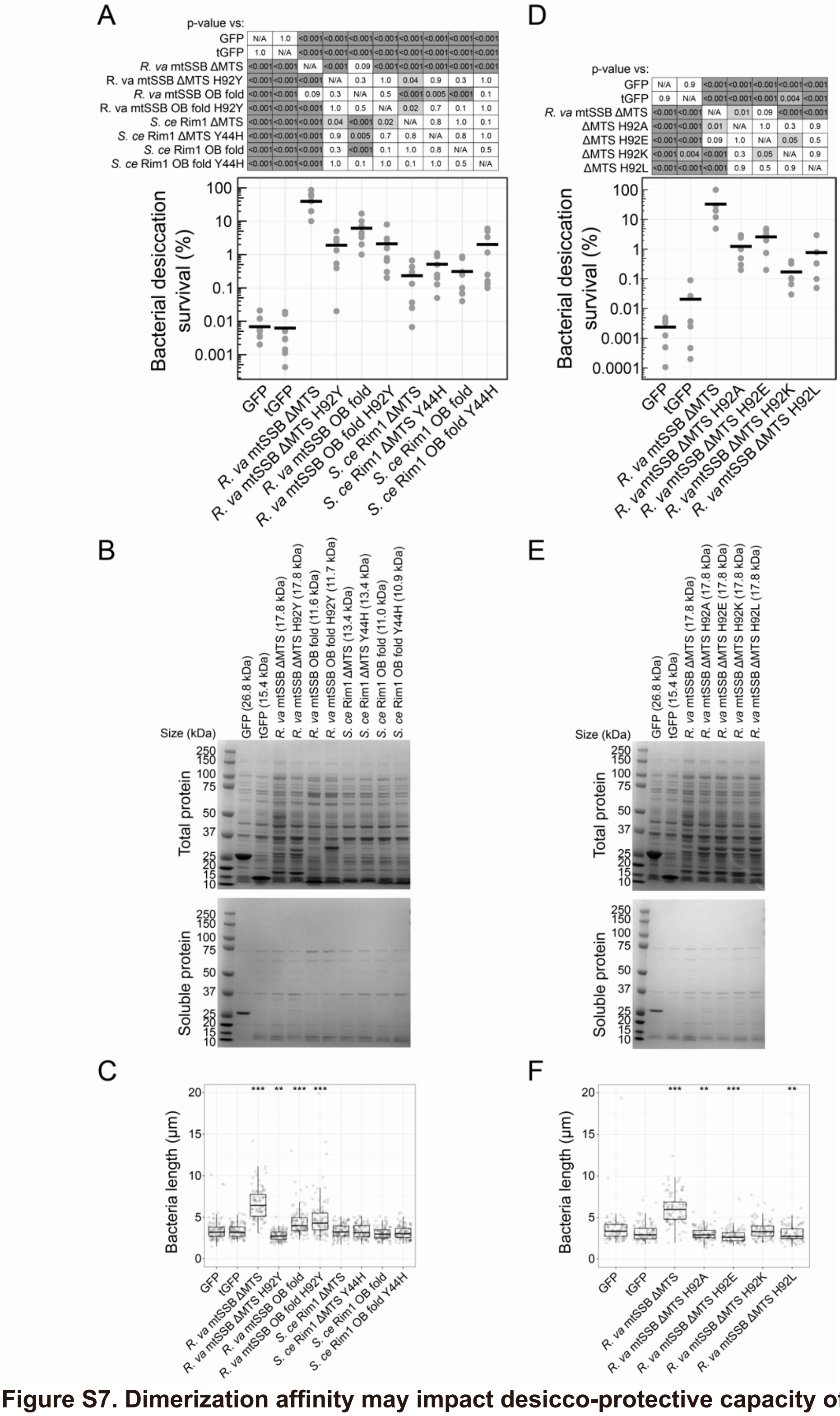

Figure S7. Dimerization affinity may impact desicco-protective capacity of mtSSBs.
A) Amino acid substitutions replaced histidine 92 of *R. varieornatus* mtSSB or its OB fold with the tyrosine of Rim1. Similarly, tyrosine 44 of Rim1 was swapped for a histidine. Desiccation survival of bacteria expressing these constructs is shown. **B)** Protein expression and solubility levels of proteins with amino acid substitutions were determined with SDS-PAGE. **C)** Length of bacteria expressing the same constructs as in A and B is plotted. **D)** Bacterial desiccation survival is

diminished in strains expressing *R. varieornatus* mtSSB with mutations in H92 that are likely to disrupt its dimerization. **E)** Protein gels show total and soluble protein from bacteria expressing *R. varieornatus* mtSSB with point mutations likely to disrupt its ability to dimerize. **F)** Bacterial length is plotted for bacteria expressing the constructs in D and E. P-values in A and D were calculated with Tukey tests. P-values in C and F were calculated with Dunnett's tests. ** $p < 0.01$, *** $p < 0.001$.

p-value vs:								
GFP (SV)	N/A	1.0	0.9	0.9	0.008	<0.001	<0.001	<0.001
GFP (chamber)	1.0	N/A	1.0	1.0	0.03	<0.001	<0.001	<0.001
tGFP (SV)	0.9	1.0	N/A	1.0	0.1	0.003	<0.001	<0.001
tGFP (chamber)	0.9	1.0	1.0	N/A	0.1	0.003	<0.001	<0.001
R. va mtSSB (SV)	0.008	0.03	0.1	0.1	N/A	0.8	0.1	0.1
R. va mtSSB (chamber)	<0.001	<0.001	0.003	0.003	0.8	N/A	0.8	0.8
R. va mtSSB ΔMTS (SV)	<0.001	<0.001	<0.001	<0.001	0.1	0.8	N/A	1.0
R. va mtSSB ΔMTS (chamber)	<0.001	<0.001	<0.001	<0.001	0.1	0.8	1.0	N/A

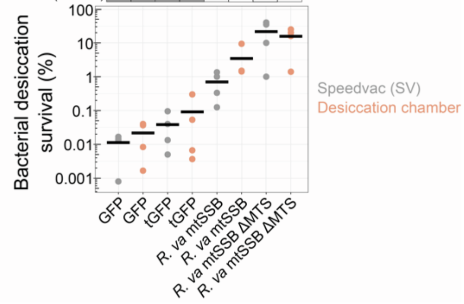

Figure S8. Bacterial desiccation survival is similar independent of the method of drying. Desiccation survival is plotted for *E. coli* expressing GFP, truncated GFP, *R. va* mtSSB, or *R. va* mtSSB ΔMTS. Bacteria were either desiccated overnight in a speedvac (gray) or in a desiccation chamber with desiccant (orange). Bars represent mean values and p-values were calculated with a Tukey test following a significant 1-way ANOVA result ($p<0.001$, $n=4$).

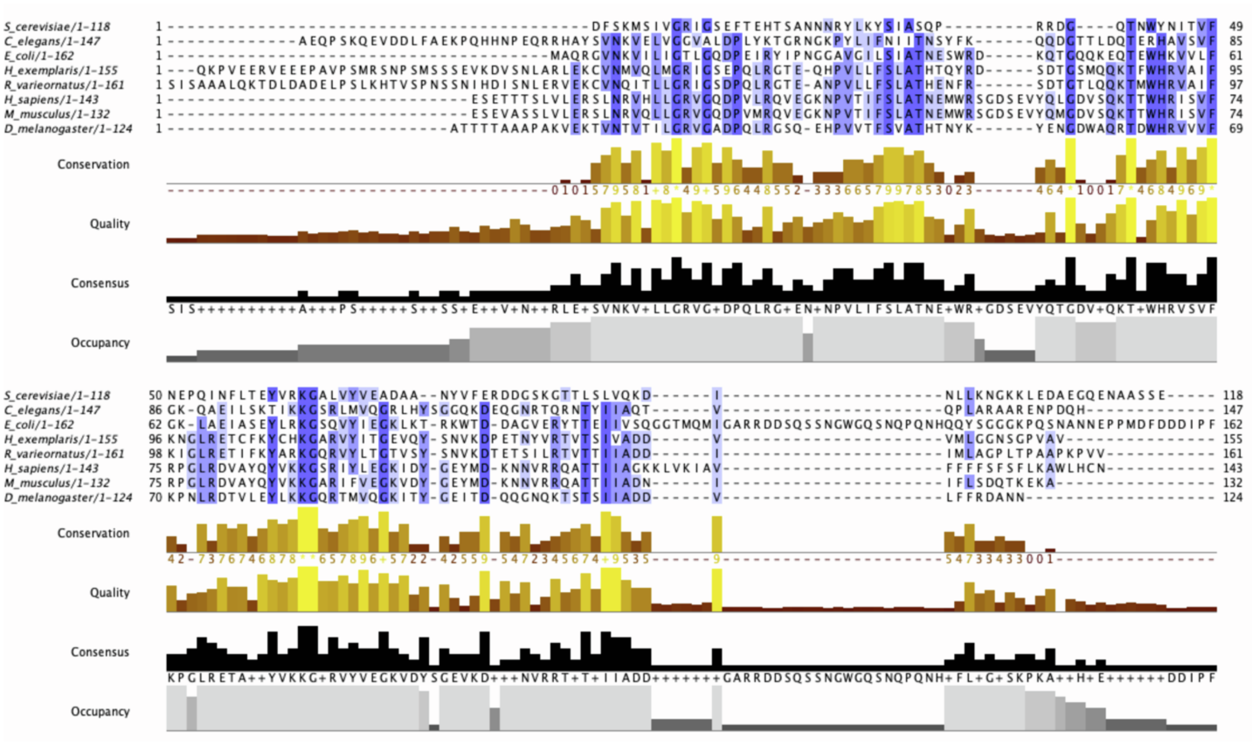

Alignment S1. Sequence alignment of mtSSBs from various organisms as well as *E. coli* SSB. Mitochondrial targeting sequences were removed from mtSSBs. Alignment was conducted with MUSCLE and visualized with JalView.
